# CD8^+^GZMK^+^CD27^+^CCR7^+^ T cells mobilized by splenic sympathetic nerves aggravate brain ischemia‒reperfusion injury via CCL19-positive endothelial cells

**DOI:** 10.1038/s41423-025-01311-9

**Published:** 2025-07-14

**Authors:** Ying Bai, Hui Ren, Shuo Leng, Mengqin Yuan, YiXin Jiang, Shenyang Zhang, Yu Wang, Minzi Ju, Zhi Wang, Wen Xi, Lian Xu, Bingjing Zheng, Daxing Li, Xinchen Huo, Tianhao Zhu, Beicheng Zhang, Ling Shen, Yuan Zhang, Wei Jiang, John H. Zhang, Bing Han, Honghong Yao

**Affiliations:** 1 Department of Pharmacology, Jiangsu Provincial Key Laboratory of Critical Care Medicine, School of Medicine, Southeast University, Nanjing, China; 2 Center of Interventional Radiology and Vascular Surgery, Department of Radiology, Zhongda Hospital, Medical School, Southeast University, Nanjing, China; 3 Department of Biomedical Engineering, Nanjing University of Aeronautics and Astronautics, Nanjing, China; 4 Shanghai Frontiers Science Center of TCM Chemical Biology, Institute of Interdisciplinary Integrative Medicine Research, Shanghai University of Traditional Chinese Medicine, Shanghai, China; 5 Department of Neurology, Affiliated Hospital of Xuzhou Medical University, Xuzhou, China; 6 Fujian Key Laboratory of Precision Medicine for Cancer, The First Affiliated Hospital, Fujian Medical University, Fuzhou, China; 7 Department of Physiology, School of Medicine, Loma Linda University, Loma Linda, CA USA; 8 Nanjing Pukou People’s Hospital, Liangjiang Hospital, Southeast University, Nanjing, China; 9 Coinnovation Center of Neuroregeneration, Nantong University, Nantong, China; 10 Institute of Life Sciences, Key Laboratory of Developmental Genes and Human Disease, Southeast University, Nanjing, China; 11 Center for Global Health, School of Public Health, Nanjing Medical University, Nanjing, China

**Keywords:** Splenic sympathetic denervation, Acute ischemic stroke, Stroke-associated T cells, CCR7, CCL19, Functional recovery, Chemokines, Acute inflammation, Mechanisms of disease

## Abstract

Splenic sympathetic activity critically modulates peripheral immunity after ischemic stroke, thus intervention in spleen sympathetic activity represents a promising therapeutic strategy for stroke. However, the mechanisms underlying spleen-brain-immune axis communication remain poorly understood. Here, we utilized a surgical denervation protocol to perform splenic sympathetic denervation (SDN), which significantly attenuated brain injury following stroke. Through single-cell RNA sequencing, we identified a novel GZMK^+^CD8^+^CD27^+^CCR7^+^ T-cell subset in patients with acute ischemic stroke (AIS), which we designated stroke-associated T (Tsa) cells. The expansion of Tsa cells was positively correlated with the severity of clinical symptoms and was driven by the splenic sympathetic nervous system. Stroke-induced sympathetic activation triggers the release of splenic norepinephrine (NE), which preferentially signals through ADRB2 on Tsa cells to promote their mobilization. Additionally, ischemic injury induces endothelial cell-specific expression of CCL19, which chemoattracts Tsa cells into the brain parenchyma via their cognate CCR7 receptor, exacerbating neuroinflammatory injury and neurological deficits in a transient middle cerebral artery occlusion (tMCAO) mouse model. We developed a CCR7-targeting peptide to disrupt this chemotactic axis and reduce T-cell infiltration, thereby mitigating brain injury. Our findings highlight SDN as a promising therapeutic strategy to attenuate ischemia‒reperfusion injury and suggest its potential as an adjunctive therapy for reperfusion treatment in AIS patients.

## Introduction

The spleen, a central organ for systemic immune responses, acts as a reservoir for various immune cells and is thought to be the first link between the central nervous system (CNS) and the peripheral immune system after stroke [1, 2]. Clinical studies have reported that approximately 40% of acute ischemic stroke (AIS) patients experience a significant reduction in splenic volume, with those showing splenic contraction exhibiting increased percentages of blood lymphocytes and elevated levels of inflammatory cytokines in their bloodstream [3, 4]. Mounting evidence has shown that splenic-derived inflammatory cells are released and infiltrate the ischemic brain to act as proinflammatory agents and exacerbate brain injury [5]. Modulation of splenic activity has emerged as a promising therapeutic target, with the potential to mitigate brain inflammatory injury and improve outcomes following AIS [6, 7].

Splenectomy, splenic pulse ultrasound treatment, and splenic irradiation have been widely used for spleen suppression and have been shown to be neuroprotective in animal studies after stroke [7–9]. However, these approaches are largely restricted in clinical practice because of the permanent elimination of splenic function, which has irreversible consequences for the immune status and overall health of patients [10]. Endovascular denervation, including endovascular splenic sympathetic denervation (SDN), has demonstrated promising results in animal models because of its efficacy, safety, and feasibility [11, 12]. Consequently, it is currently under consideration for clinical trials targeting inflammatory conditions. Further studies have confirmed that splenic sympathetic nerve signaling plays a critical role in regulating innate and adaptive immune responses following stroke [13, 14]. Nevertheless, the neuroprotective capacity of SDN against stroke-induced brain injury and its mechanistic underpinnings have not yet been fully elucidated.

The function of the immune response in AIS has garnered increasing attention, with T lymphocytes emerging as pivotal mediators of both detrimental and reparative immune responses [15–17]. Accumulating evidence supports multifaceted roles of T lymphocytes in response to cerebral ischemia [18, 19]. Although previous studies established the infiltration of peripheral T cells into the ischemic brain as a hallmark of poststroke immune activation [18], recent advances have further delineated the nuanced contributions of different T-cell subsets. CD4^+^ helper T cells secrete IFN-γ and IL-21, and deficiency of these cytokines significantly attenuates cerebral infarct volume in experimental stroke models [20, 21]. CD8^+^ cytotoxic T lymphocytes (CTLs) amplify neurotoxicity through granzyme B and perforin release [22], whereas regulatory T cells (Tregs) promote white matter repair by activating microglial reparative pathways via osteopontin–integrin signaling [17]. These updates underscore the complex interplay of T-cell subsets in AIS pathophysiology; however, whether SDN affects immune cell alterations remains unknown.

In this study, we employed a surgical denervation protocol to perform SDN in the spleen of a stroke mouse model and reported that SDN effectively reduces ischemia‒reperfusion injury in the context of stroke. Next, we performed an unbiased analysis of single-cell RNA sequencing (scRNA-seq) data from peripheral blood mononuclear cells (PBMCs) from AIS patients before and after reperfusion therapy. We identified a previously unrecognized GZMK^+^CD8^+^CD27^+^CCR7^+^ T-cell subset in AIS patients, named stroke-associated T (Tsa) cells, which was significantly positively correlated with the severity of clinical symptoms. Stroke-induced sympathetic activation triggers the release of splenic norepinephrine (NE), which selectively signals through β2-adrenergic receptors (ADRB2) on Tsa cells to drive their mobilization. Subsequent brain infiltration of Tsa cells is orchestrated by CCL19 derived from a specific endothelial cell subset after stroke via the CCL19/CCR7 axis. Consequently, intracerebral Tsa cells amplified neuroinflammation, a pathogenic cascade effectively abrogated by CCR7-targeting peptide inhibitors. Our study illuminates SDN as a clinically feasible strategy to optimize reperfusion outcomes while identifying CCR7 inhibition as a parallel therapeutic avenue.

## Results

### Sympathetic denervation of the spleen reduces brain injury after ischemia‒reperfusion

**Fig. 1:**
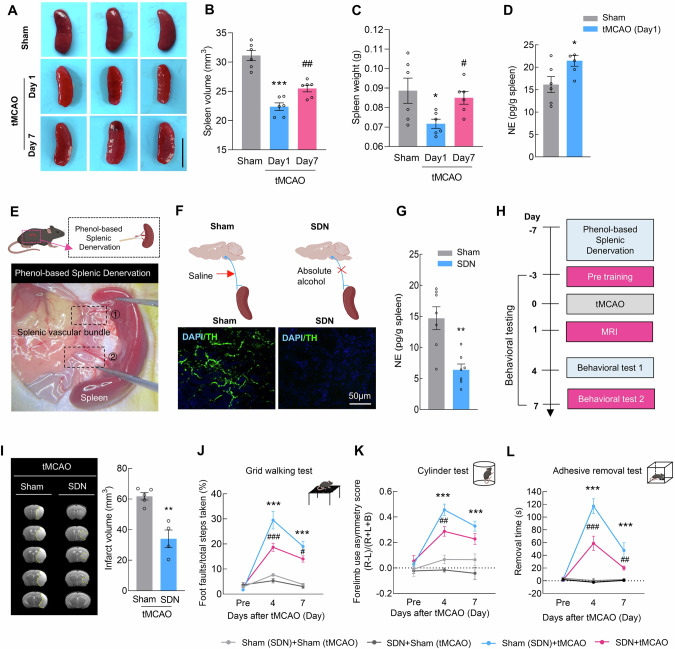
Sympathetic denervation of the spleen reduces brain injury after ischemia‒reperfusion. **A** Spleen images of the sham and tMCAO models during the early (day 1) and late (day 7) stages. The scale bar represents 1 cm. **B** Spleen volume in the sham and tMCAO models during the early (day 1) and late (day 7) stages. *n* = 6/group. **C** Spleen weight in the sham and tMCAO models during the early (day 1) and late (day 7) stages. *n* = 6/group. **D** ELISA-based measurement of splenic norepinephrine (NE) levels at 24 h post tMCAO. *n* = 6/group. **E** Schematic illustration of the splenic sympathetic denervation (SDN) experiment. **F** Representative images of spleen sections from sham-operated or denervated mice 1 week after surgery. Green, tyrosine hydroxylase (TH) staining; blue, DAPI. Scale bar = 50 μm. **G**. NE concentrations in splenic tissue from sham-operated and denervated mice. *n* = 7/group. **H** Schematic of tMCAO, infarction examination, and behavioral tests in SDN mice. **I** Representative T2-weighted MR images of the tMCAO (sham) and tMCAO (SDN) groups 1 day after tMCAO, followed by analysis of the infarct volume. The dashed line denotes the infarct area. *n* = 5 in the tMCAO (Sham) group, *n* = 4 in the tMCAO (SDN) group. Functional recovery in SDN mice was assessed by the grid walking test (**J**), cylinder test (**K**), and adhesive removal test **(L**) at baseline and on days 4 and 7 after tMCAO. *n* = 6/group. The data are presented as the means ± SEMs. *P < 0.05; **P < 0.01; ***P < 0.001; ^#^*P* < 0.05^; ##^*P* < 0.01; ^###^*P* < 0.001

Splenic atrophy is an important feature of the stroke-induced systemic inflammatory response and immunomodulatory disorders and is characterized by a reduced organ size that occurs within the first few days after stroke [23]. Compared with those of the sham controls, the spleen volume and weight were significantly lower on day 1 after transient middle cerebral artery occlusion (tMCAO) and recovered on day 7 (Fig. 1A–C). Quantitative analysis revealed that the levels of NE in the spleen were significantly increased at 1 day post-stroke (Fig. 1D). Considering that the dominant nerves innervating the spleen are postganglionic fibers of the abdominal sympathetic nervous system, we employed a surgical denervation protocol to perform SDN in the spleen (Fig. 1E). Analysis of the tyrosine hydroxylase (TH) and NE levels in the spleen confirmed successful sympathetic nerve denervation (Fig. 1F, G).

Next, we examined the effect of SDN on brain damage after stroke (Figs. 1H and S1A, B). Cerebral blood flow analysis indicated that SDN did not affect brain blood flow during tMCAO modeling (Fig. S1C, D). MRI analysis revealed that the infarct volume was significantly reduced in tMCAO mice that received SDN surgery (Fig. 1I). For the grid-walking task, SDN significantly decreased the rate of foot faults in comparison with that in mice that did not undergo SDN surgery on day 4 and 7 after tMCAO model induction (Fig. 1J). Mice that were subjected to SDN surgery showed significantly reduced bias in the cylinder test on day 4 and 7 after tMCAO model induction (Fig. 1K). A significant recovery effect was also observed in the adhesive removal test, reflected by the decreased removal time after SDN surgery compared with that in the sham (SDN) group on day 4 and 7 after tMCAO model induction (Fig. 1L). These results suggest that SDN may effectively reduce brain injury caused by tMCAO, highlighting its potential as a therapeutic intervention after stroke.

### Cell subset-specific analysis identifies homeostatic and stroke-associated CD8^+^GZMK^+^ T cells

**Fig. 2:**
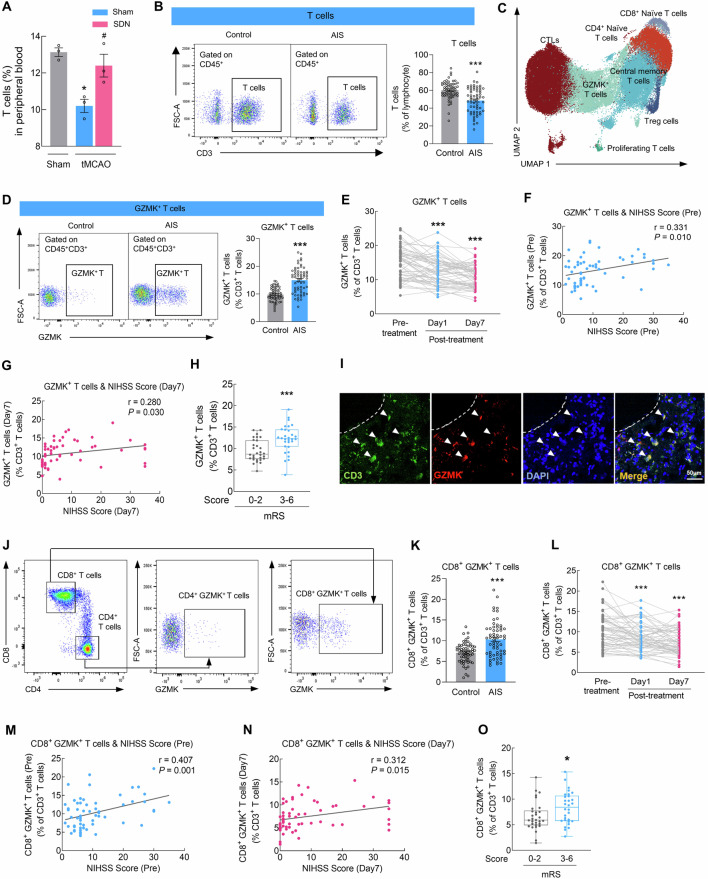
Cell subset-specific analysis identifies homeostatic and stroke-associated GZMK^+^ CD8^+^ T cells. **A** Proportions of T cells in peripheral PBMCs after SDN in sham and tMCAO mice. **B** Proportions of T cells in non-stroke controls and AIS patients. *n* = 60/group. **C** UMAP visualization showing 7 transcriptionally distinct T-cell subsets. **D** Proportions of GZMK^**+**^ T cells in non-stroke controls and AIS patients. *n* = 60/group. **E** Proportions of GZMK^**+**^ T cells in AIS patients before treatment, on day 1, and on day 7 after treatment. *n* = 60/group. **F** Spearman correlation between GZMK^**+**^ T-cell proportions and National Institutes of Health Stroke Scale (NIHSS) scores (pre-treatment). **G** Spearman correlation between GZMK^**+**^ T-cell proportions and NIHSS scores (day 7 post-treatment). **H** Proportions of GZMK^**+**^ T cells in patients with good outcomes (mRS 0–2) versus poor outcomes (mRS 3–6) post-treatment; *n* = 30 in the good outcome group and *n* = 30 in the poor outcome group. **I** Representative images showing immunostaining for CD3 (green) and GZMK (red) in the brains of AIS patients. **J** Gating strategy for determining the abundance of CD4^**+**^ GZMK^**+**^ T cells and CD8^**+**^ GZMK^**+**^ T cells via flow cytometry. **K** Proportions of CD8^**+**^ GZMK^**+**^ T cells in non-stroke controls and AIS patients. *n* = 60/group. **L** Proportions of CD8^**+**^ GZMK^**+**^ T cells in AIS patients before treatment, on day 1, and on day 7 after treatment. *n* = 60/group. **M** Spearman correlation between CD8^**+**^ GZMK^**+**^ T-cell proportions and National Institutes of Health Stroke Scale (NIHSS) scores (pre-treatment). **N** Spearman correlation between CD8^**+**^ GZMK^**+**^ T-cell proportions and NIHSS scores (day 7 post-treatment). **O** Proportions of CD8^**+**^ GZMK^**+**^ T cells in patients with good outcomes (mRS 0–2) versus poor outcomes (mRS 3–6) post-treatment; *n* = 30 in the good outcome group and *n* = 30 in the poor outcome group. The data are presented as the means ± SEMs. *P < 0.05; ***P < 0.001

The postganglionic fibers of the abdominal sympathetic nervous system that innervate the spleen primarily run along the splenic artery, with the surrounding area consisting mainly of lymphoid sheaths (T-cell zones) [24]. Thus, we assessed the effects of SDN on T cells and found that SDN surgery reversed the decrease in T-cell frequency in tMCAO mice (Fig. 2A). These results indicate that SDN can alter the immune status of T cells after stroke. To validate these findings in clinical patients, we established a cohort comprising 60 AIS patients who underwent recanalization therapy and 60 non-stroke controls. Flow cytometric analysis of PBMCs revealed that in AIS patients, the proportion of T cells among PBMCs was significantly lower than that in non-stroke controls (Fig. 2B and Table S1).

To further clarify the relationship between T-cell immunity after stroke and clinical characteristics, we generated a scRNA-seq library from 3 AIS patients before and after stroke (day 1 and day 7) who received mechanical thrombectomy combined with IV recombinant tissue plasminogen activator (rt-PA) thrombolysis treatment in PBMCs, along with 3 controls matched for sex and age (Figs. S2, 3, S4A, and Table S2). Unbiased, graph-based clustering identified four major cell populations: T cells, B cells, myeloid cells, and NK cells (Fig. S4B–D). We captured T cells and subclustered them into seven subsets according to previously defined marker genes, encompassing well-defined subsets—CD4^+^ naive T cells (*CD4*, *SELL*, *TCF7*, and *LEF1*) [25], CD8^+^ naive T cells (*CD8*, *SELL*, *TCF7*, and *LEF1*) [25], proliferating T cells (*MKI67*, *TYMS*, and *PCLAF*) [26, 27], GZMK^+^ T cells (*GZMK*, *CCR5*, and *CXCR6*) [26, 28], central memory T (T_CM_) cells (*AQP3*, *CPR183*, and *IL7R*) [29–31], Treg cells (*FOXP3*, *IKZF2*, and *IL2RA*) [32], and cytotoxic T cells (CTLs) (*GZMB*, *GNLY*, and *FGFBP2*) [26, 33]—each characterized by unique signature genes and expression profiles (Figs. 2C, S5A, B and S6A–C). Flow cytometry analysis revealed increased counts of GZMK^+^ T cells and CTLs and downstream CD4^+^ naive T cells, T_CM_ cells, and Tregs in the PBMCs of AIS patients (Figs. 2D, S7, and S8A). The frequencies of naive CD4^+^ T cells, T_CM_ cells, Treg cells, GZMK^+^ T cells, and CTLs were changed on day 1 and 7 after reperfusion therapy (Figs. 2E and S8B). However, only the GZMK^+^ T-cell frequency was positively correlated with the National Institutes of Health Stroke Scale (NIHSS) score (*r* = 0.331, *P* = 0.010) in AIS patients before reperfusion therapy (Figs. 2F and S8C) and negatively correlated with the Barthel Index (BI) score (pre-treatment) (*r* = -0.304, *P* = 0.018) (Fig. S9A), suggesting that the GZMK^+^ T-cell frequency is associated with patient clinical severity.

Moreover, on day 7 post-treatment, although the frequency of GZMK^+^ T cells in AIS patients significantly recovered along with their symptoms, GZMK^+^ T cells remained positively correlated with the NIHSS score (*r* = 0.280, *P* = 0.030) and negatively correlated with the BI score (*r* = −0.468, *P* < 0.001) (Figs. 2G and S9B). We further verified whether the proportions of GZMK^+^ T cells were associated with stroke outcome. A Mann‒Whitney *U* test revealed a significant difference in GZMK^+^ T-cell frequencies on day 7 post-treatment between patients who eventually achieved good outcomes (mRS 0–2) and those with poor outcomes (mRS 3–6) (Fig. 2H). Importantly, immunofluorescence staining revealed infiltration of GZMK^+^ T cells into the peri-infarct area of the brain of AIS patients (Fig. 2I), suggesting that these cells may directly regulate CNS pathology by infiltrating the brain parenchyma.

GZMK^+^ T cells can be subdivided into CD4^+^ and CD8^+^ subsets. Flow cytometry analysis revealed that before treatment, the proportions of both CD4^+^ GZMK^+^ and CD8^+^ GZMK^+^ T cells were greater in the PBMCs of AIS patients than in those of non-stroke controls (Figs. 2J, K and S10A). After reperfusion, the proportions of CD8^+^ GZMK^+^ T cells, but not those of CD4^+^ GZMK^+^ T cells, were significantly lower on days 1 and 7 than they were before reperfusion (Figs. 2L and S10B). CD8^+^ GZMK^+^ T cells were positively correlated with the NIHSS score and negatively correlated with the BI before and after reperfusion therapy on day 7 (Figs. 2M, N and S10C, D), whereas CD4^+^ GZMK^+^ T cells were not correlated with the severity of AIS (Fig. S10E–H). To further compare the value of these two types of cells for prognostic assessment in AIS patients, we compared their proportions in AIS patients with good outcomes (mRS 0–2) to those with poor outcomes (mRS 3–6). A greater proportion of CD8^+^ GZMK^+^ T cells, but not CD4^+^ GZMK^+^ T cells, was observed in AIS patients with poor outcomes on day 7 post-treatment (Figs. 2O and S10I). These analyses underscore the distinctive characteristics of different GZMK^+^ T-cell subsets, emphasizing that CD8^+^ GZMK^+^ T cells, rather than CD4^+^ GZMK^+^ T cells, are associated with ischemic stroke and reperfusion.

### CCR7-specific CD8^+^ GZMK^+^ T cells are associated with clinical symptoms in AIS patients

**Fig. 3:**
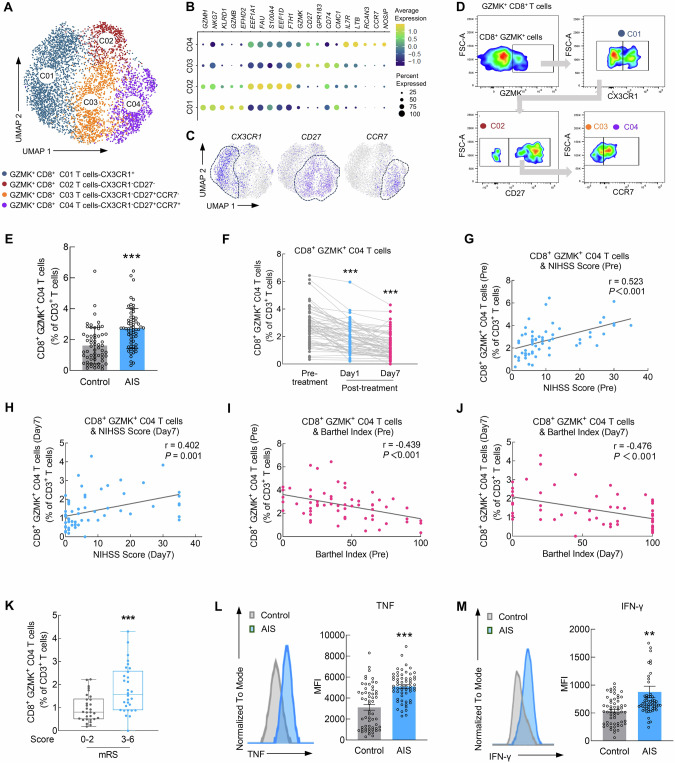
CCR7-specific CD8^+^ GZMK^+^ T cells are associated with clinical symptoms in AIS patients. **A** UMAP visualization showing four transcriptionally distinct CD8^**+**^ GZMK^**+**^ T-cell subsets. **B** Dot plot showing the expression of the five selected genes in each subtype. **C** Feature plots showing the gene expression of representative markers across four subsets. **D** Gating strategy for determining the abundance of four CD8^**+**^ GZMK^**+**^ T-cell sub-populations via flow cytometry. **E** Proportions of CD8^**+**^ GZMK^**+**^ C04 T cells in non-stroke controls and AIS patients. *n* = 60/group. **F** Proportions of CD8^**+**^ GZMK^**+**^ C04 T cells in AIS patients before treatment, on day 1, and on day 7 after treatment. *n* = 60/group. **G** Spearman correlation between CD8^**+**^ GZMK^**+**^ C04 T-cell proportions and National Institutes of Health Stroke Scale (NIHSS) scores (pre-treatment). **H** Spearman correlation between CD8^**+**^ GZMK^**+**^ C04 T-cell proportions and NIHSS scores (day 7 post-treatment). **I** Spearman correlation between CD8^**+**^ GZMK^**+**^ C04 T-cell proportions and BI scores (pre-treatment). **J** Spearman correlation between CD8^**+**^ GZMK^**+**^ C04 T-cell proportions and BI scores (day 7 post-treatment). **K** Proportions of CD8^**+**^ GZMK^**+**^ C04 T cells in patients with good outcomes (mRS 0–2) versus poor outcomes (mRS 3–6) before treatment; n = 30 in the good outcome group and *n* = 30 in the poor outcome group. **L** Quantification of TNF in non-stroke controls and AIS patients. *n* = 60/group. **M** Quantification of IFN-γ in non-stroke controls and AIS patients. *n* = 60/group. The data are presented as the means ± SEMs. **P < 0.01; ***P < 0.001

We next focused on CD8^+^ GZMK^+^ T cells to understand the specific T-cell response to ischemic injury and reperfusion treatment. Upon unsupervised reclustering, CD8^+^ GZMK^+^ T cells were divided into four distinct subsets: CD8^+^GZMK^+^CX3CR1^+^ T cells (C01), CD8^+^GZMK^+^CX3CR1^−^CD27^−^ T cells (C02), CD8^+^GZMK^+^CX3CR1^−^CD27^+^CCR7^−^ T cells (C03), and CD8^+^GZMK^+^CX3CR1^−^CD27^+^CCR7^+^ T cells (C04) (Fig. 3A–C). Flow cytometry analysis revealed increased counts of C02, C03, and C04 CD8^+^ GZMK^+^ T cells and unchanged C01 CD8^+^ GZMK^+^ T cells in the PBMCs of AIS patients (Figs. 3D, E and S11A–C). However, on day 1 and 7 after AIS patients received reperfusion therapy, there were no significant changes in the frequencies of the C01 and C02 subsets, whereas the frequencies of the C03 and C04 subsets significantly decreased (Figs. 3F and S11D–F). On the basis of the analysis of the four CD8^+^ GZMK^+^ T subsets as well as the NIHSS and BI scores before and after treatment, we identified significant positive correlations between C04 subset frequencies and NIHSS scores in AIS patients both before (*r* = 0.523, *P* < 0.001) and after treatment (*r* = 0.402, *P* = 0.001) (Fig. 3G, H). Conversely, negative correlations were observed between the frequencies of the C04 subset and BI scores both pre- (*r* = −0.439, *P* < 0.001) and post-treatment (*r* = −0.476, *P* < 0.001) (Fig. 3I, J). The C01, C02, and C03 subsets did not correlate with either the NIHSS score or BI score in AIS patients before or after treatment (Fig. S12A–D). Additionally, on day 7 post-treatment, the proportions of CD8^+^ GZMK^+^ C04 T cells among total T cells were significantly different between AIS patients who eventually achieved good outcomes (mRS 0–2) and those with poor outcomes (Fig. 3K), revealing that CD8^+^ GZMK^+^ C04 T cells were associated with stroke outcome. Interestingly, the levels of proinflammatory cytokines (TNF and IFN-γ) in CD8^+^ GZMK^+^ C04 T cells were significantly greater in AIS patients than in controls (Fig. 3L‒M). Therefore, CD8^+^ GZMK^+^ C04 T cells, which we named Tsa cells, attracted our attention in our subsequent studies.

### Adrenergic innervation promotes splenic generation of Tsa cells after reperfusion in stroke model mice

The spleen actively participates in cellular immunity and regulates the frequency of T-cell subsets in the peripheral blood [34]. In response to external or internal stimuli, immune cells become activated and differentiate into populations of effector cells with multiple functions. To track the source of Tsa cells, we performed differentiation tracing of CD8^+^ naive T cells, CD8^+^ T_CM_ cells, and CD8^+^ T_EM_ cells in the spleen, blood, and brain in tMCAO stroke model mice via flow cytometry. As crucial secondary lymphoid organs, the spleen functions in the recirculation and residency of T cells. In the spleen, CD8^+^ naive T cells and CD8^+^ T_CM_ cells were significantly decreased, whereas CD8^+^ T_EM_ cells and Tsa cells were increased after reperfusion in tMCAO mice (Fig. S13A). In peripheral PBMCs, CD8^+^ T_CM_, total CD8^+^ T_EM_, and Tsa cells were significantly increased after reperfusion, but the frequency of CD8^+^ naive T cells was decreased (Fig. S13B). We next examined the infiltration of these cells in the stroke brain. As shown in Fig. S13C, the majority of infiltrating CD8^+^ T cells consisted of total T_EM_ and Tsa cells, both of which were significantly increased in the peri-infarct region of tMCAO mice. During this process, Tsa cells undergo a marked proliferative phase, rapidly migrating toward the peri-infarct area of the brain. Taken together, our findings revealed that the trend of changes in Tsa cells remained consistent, peaking at 24 h and subsequently plateauing from 24–72 h.

**Fig. 4:**
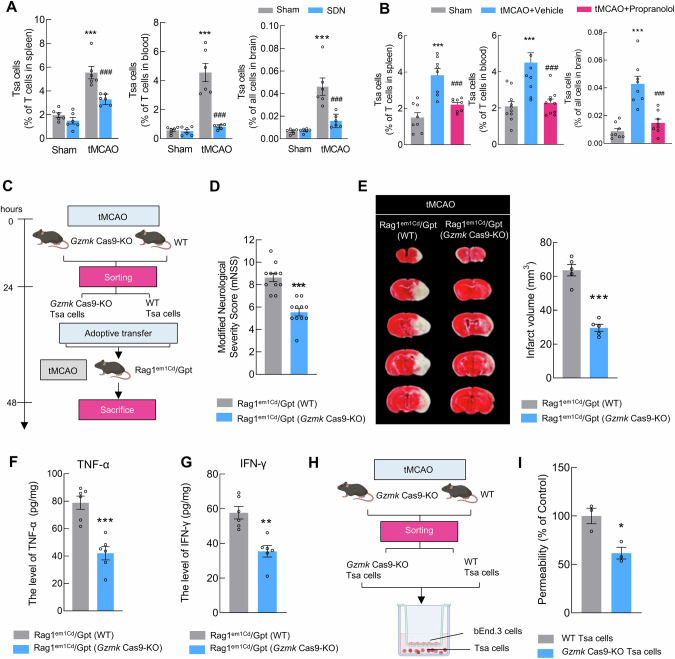
Adrenergic innervation promotes splenic generation of Tsa cells after reperfusion in stroke mice. **A** Proportions of Tsa cells in the spleen, blood, and brain after SDN in sham and tMCAO mice. The proportions of Tsa cells were determined by flow cytometry 24 h after reperfusion. *n* = 6/group. **B** Proportions of Tsa cells in the spleen, blood, and brain 24 h after tMCAO in the vehicle and propranolol groups. **C** Isolation and adoptive transfer strategy for Tsa cells from WT and *Gzmk* Cas9-KO mice. **D** Modified neurological severity score of Rag1^em1Cd^/Gpt mice that received Tsa cells isolated from WT mice and those that received Tsa cells isolated from *Gzmk* Cas9-KO mice at 24 h after stroke. *n* = 11/group. **E** Infarct volume of Rag1 ^em1Cd^/Gpt mice that received Tsa cells isolated from WT mice and those that received Tsa cells isolated from *Gzmk* Cas9-KO mice at 24 h after stroke. *n* = 5/group. **F**, **G** The levels of the inflammatory factors TNF-α and IFN-γ in the peri-infarct area of Rag1 ^em1Cd^/Gpt mice that received Tsa cells isolated from WT mice and the group that received Tsa cells isolated from *Gzmk* Cas9-KO mice at 24 h after stroke. *n* = 6/group. **H**, **I** In vitro endothelial barrier permeability assay using Tsa cells from WT and *Gzmk*-KO mice. *n* = 3/group. The data are presented as the means ± SEMs. *P < 0.05; **P < 0.01; ***P < 0.001; ^###^*P* < 0.001

The major neurogenic signal influencing T-cell differentiation is catecholamine via adrenergic innervation. The dominant nerves innervating the spleen are postganglionic fibers of the abdominal sympathetic nervous system. Thus, we conducted SDN in the spleen. On day 7 after SDN, the mice were subjected to sham or tMCAO surgery. We found that SDN markedly suppressed the reperfusion-induced increase in Tsa cells in the spleen, peripheral PBMCs, and brain (Fig. 4A), indicating that the expansion of Tsa cells was driven by the splenic sympathetic nervous system. Additionally, we analyzed the profiles of different receptors via scRNA-seq and revealed the predominant expression of ADRB2 (β2-adrenergic receptor) (Fig. S14A). ADRB2 was highly expressed in these cells, as validated by sorting Tsa cells via qPCR (Fig. S14B). Importantly, pharmacological blockade with propranolol significantly attenuated Tsa cell generation (Fig. 4B). These findings collectively demonstrate that stroke triggers sympathetic neurons to release NE in the spleen, which preferentially signals through ADRB2 on Tsa cells to drive their mobilization.

To characterize Tsa cells further, we performed weighted gene coexpression network analysis (WGCNA) combined with GO enrichment and KEGG analyses to conduct an in-depth exploration of Tsa cell characteristics (Fig. S15). GO analysis revealed that Tsa cells exhibit functional modules associated with T-cell activation and differentiation, including “T-cell activation”, “T-cell differentiation”, and “Regulation of immune system process” (Fig. S16A). Additionally, through KEGG analysis of each module, we determined that the M3 module, which is highly associated with Tsa cells, is linked to the activation of the NF-κB signaling pathway, a central mediator of inflammatory responses (Fig. S16B).

Tsa cells are a subset of CD8^+^ T cells characterized by GZMK expression. To investigate the role of Tsa cells in stroke, we utilized *Gzmk* Cas9-KO and WT mice. After sorting Tsa cells on the basis of the respective markers, we adoptively transferred them into the tMCAO model *Rag1*^em1Cd^/Gpt mice (Fig. 4C). As shown in Fig. 4D, E, *Rag1*^em1Cd^/Gpt mice that received “Tsa cells” from *Gzmk* Cas9-KO mice presented improved neurological deficit scores and decreased infarct volumes compared with those of the group that received Tsa cells isolated from WT mice. To explore the underlying mechanism, we measured the secretion of TNF-α and IFN-γ in sorted Tsa and *Gzmk* KO-Tsa cells. Compared with Tsa cells, *Gzmk* KO-Tsa cells exhibited a reduced secretory capacity for inflammatory cytokines (Fig. 4F, G). To further validate these findings, we conducted an in vitro experiment using a Transwell system. Mouse endothelial cells were seeded in the upper chamber, while Tsa cells or *Gzmk* KO-Tsa cells were cocultured in the lower chamber (Fig. 4H). Compared with Tsa cells, *Gzmk* KO-Tsa cells demonstrated a statistically significant reduction in their ability to disrupt endothelial cell permeability, as evidenced by diminished fluorescence leakage, indicating their impaired capacity to compromise the blood‒brain barrier (Fig. 4I).

### Identification of the stroke-associated endothelial cell subset that mediates Tsa cell infiltration

**Fig. 5:**
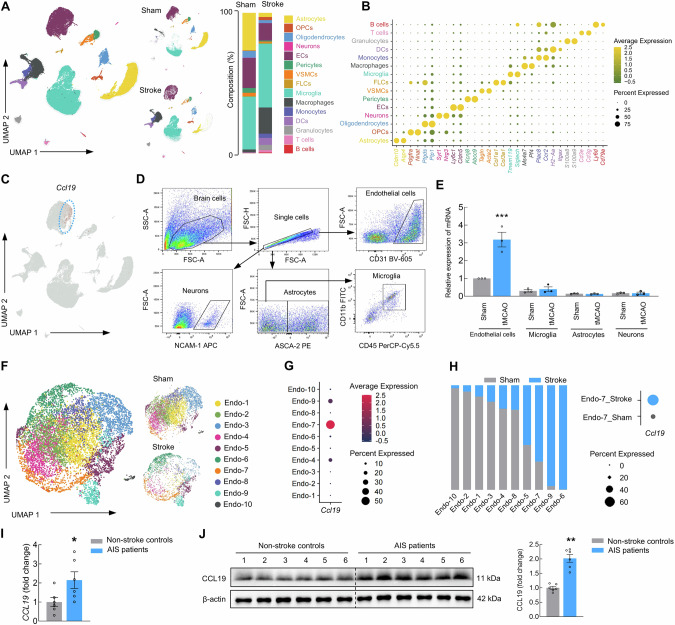
Identification of the stroke-associated endothelial cell subset that mediates Tsa cell infiltration. **A** UMAP of the 15 major cell populations identified in the peri-infarct cortex in the stroke group and the corresponding regions in the sham group. Left: Integration of data from the sham and stroke groups. Top middle: the sham group. Bottom middle: The stroke group. Right: Stacked bar graphs showing the proportions of different cells in the sham and stroke groups. **B** Cell type annotation was performed on the basis of the expression of well-established marker genes. The color of the dots indicates the expression level. The dot size represents the proportion of cells expressing the indicated markers. **C** Feature plot of *Ccl19* expression in all cells. **D** Schematic of endothelial cells, microglia, astrocytes, and neurons isolated from the brains of tMCAO stroke model mice. **E** Relative expression of *Ccl19* in the sorted cells as determined by qPCR in the sham and tMCAO mice. *n* = 3 samples for each group; each sample was pooled from 3 animals. **F** UMAP of the 10 cell populations identified by endothelial cell clustering. Left: Integration of data from the sham and stroke groups. Top right: the sham group. Bottom right: The stroke group. **G** Dot plot showing the expression of *Ccl19* in endothelial cell subsets. **H** Bar graph showing the percentages of the various endothelial cell subtypes identified in (**F**) in both the sham and stroke groups. Dot plot showing the expression of *Ccl19* in the endo-7 subset in both the sham and stroke groups. **I** Relative expression of CCL19 in the brains of non-stroke controls and AIS patients, as determined by qPCR. *n* = 6/group. **J** CCL19 expression in the brains of non-stroke controls and AIS patients was measured via western blotting. *n* = 6/group. The data are presented as the means ± SEMs. *P < 0.05; **P < 0.01; ***P < 0.001

The recruitment of T lymphocytes into the brain parenchyma requires chemotactic cues [35]. To identify stroke-associated parenchymal cell types that may mediate Tsa cell infiltration into ischemic brains, we employed scRNA-seq in brain tissue obtained from the peri-infarct region of the stroked mouse brain. This process generated a cell-by-gene expression matrix, and dimensionality reduction was executed via UMAP and graph-based clustering, revealing a total of 15 major cell types (Fig. 5A). Dimensionality reduction revealed substantial phenotypic disparities between stroke and sham mice, and we identified each cell type on the basis of specific cell markers: astrocytes (*Cldn10* and *Aqp4*), oligodendrocyte precursor cells (OPCs) (*Pdgfra* and *Nnat*), oligodendrocytes (*Ptgds* and *Plp1*), neurons (*Syt1* and *Nrg3*), endothelial cells (*Ly6c1* and *Cldn5*), pericytes (*Kcnj8* and *Abcc9*), vascular smooth muscle cells (VSMCs) (*Tagln* and *Acta2*), fibroblast-like cells (FLCs) (*Col1a1* and *Col3a1*), microglia (*Tmem119* and *Siglech*), macrophages (*Ms4a7* and *Pf4*), monocytes (*Ccr2* and *Plac8*), dendritic cells (DCs) (*H2-Aa* and *Itgax*), neutrophils (*S100a8* and *S100a9*), natural killer (NK) and T cells (*Cd3e* and *Cd3g*), and B cells (*Ly6d* and *Cd79a*) (Fig. 5A, B).

Given that CCR7, a migration marker, is highly expressed in Tsa cells, we next analyzed the expression patterns of its ligands CCL19 and CCL21 across various cell types via scRNA-seq. We found that *Ccl19*, but not *Ccl21*, was selectively expressed in endothelial cells in our scRNA-seq data (Fig. 5C). Flow cytometry combined with qPCR analysis confirmed the increased expression of *Ccl19* in endothelial cells after stroke compared with that in the sham group (Fig. 5D, E). Intriguingly, we found that only a portion of endothelial cells expressed *Ccl19*, indicating subtype-specific expression of *Ccl19* in endothelial cells. We thus selected endothelial cells and reclustered them into ten subsets: Endo-1 to Endo-10 (Fig. 5F). Notably, we found that the proportion of Endo-7, which highly expresses the chemokine *Ccl19*, was significantly increased in the peri-infarct areas of stroke mice (Fig. 5G, H). Compared with that in the sham group, *Ccl19* was highly expressed only in the Endo-7 subset from the stroke group. Furthermore, we collected somatosensory cortex tissues from AIS patients at the Chinese Brain Bank Center (CBBC) (Wuhan, China). The demographic and clinical characteristics of the AIS patients and non-stroke controls are presented in Table S3. CCL19 was elevated in the samples from patients with AIS compared with those from non-stroke controls, as determined by qPCR and western blot analysis (Fig. 5I, J).

Additionally, to elucidate whether Tsa exhibits tissue-specific features related to organ infiltration, we utilized data from the Tabula sapiens Consortium—the most comprehensive human multiorgan single-cell transcriptomic atlas to date [36]. Our analysis revealed that in addition to being expressed in a specific subset of endothelial cells post-stroke, CCL19 exhibited low baseline expression predominantly in stromal cells under normal conditions (Fig. S17A, B). In contrast, CCL21 is expressed primarily in endothelial cells, with higher levels observed in tissues and organs, including the trachea, muscle, vasculature, and lung (Fig. S17C). To identify accumulation patterns beyond the brain, we further analyzed the enrichment of Tsa cells in various peripheral organs post-stroke via flow cytometry. Our findings indicated that Tsa cells accumulated in the lung but not in the heart or liver (Fig. S18). This tissue-specific accumulation pattern of Tsa cells suggests a potential organ-specific role for these cells in the post-stroke immune response, highlighting the complexity of systemic immune reactions following cerebral ischemia.

### CCL19-CCR7 coordinates endothelial cell-Tsa cell cross-talk

**Fig. 6:**
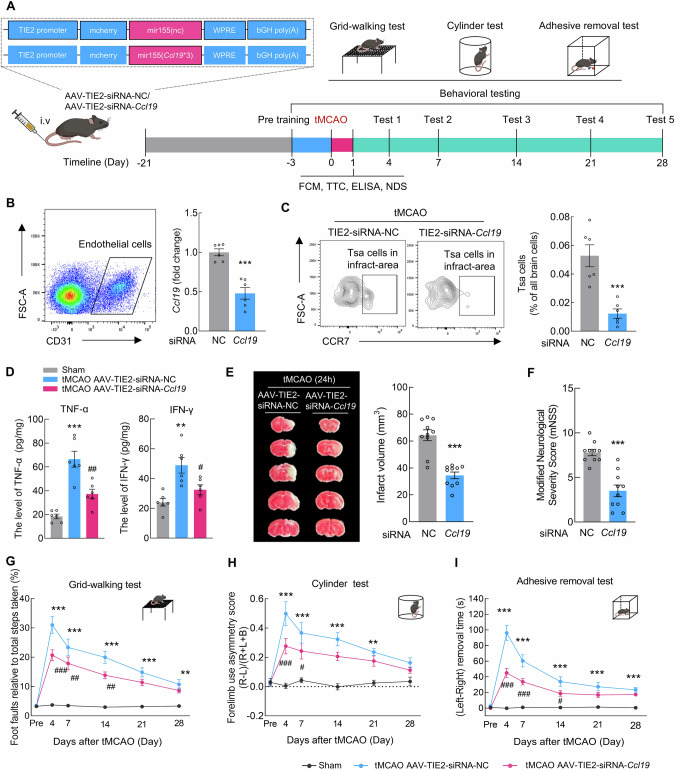
CCL19-CCR7 coordinates endothelial cell-Tsa cell cross-talk. **A** Schematic diagram of *Ccl19* lentiviral construction, AAV injection, tMCAO, flow cytometry, infarction examination, ELISA, neurological deficit assessment, and behavioral studies. **B** Endothelial cells were sorted from the brain tissues of C57BL/6J mice after AAV-TIE2-siRNA-*Ccl19* injection. The relative expression of *Ccl19* in the sorted cells was determined by qPCR. *n* = 6/group. **C** Flow cytometry analysis of the proportion of Tsa cells in the peri-infarct area of stroke mice with downregulated *Ccl19*. **D** The levels of the inflammatory factors TNF-α and IFN-γ in the peri-infarct area of tMCAO + AAV-TIE2-siRNA-NC– and tMCAO+AAV-TIE2-siRNA-*Ccl19*–injected mice were measured via ELISA 24 h after stroke. *n* = 6/group. **E** Representative TTC-stained sections from tMCAO+AAV-TIE2-siRNA-NC– and tMCAO+AAV-TIE2-siRNA-*Ccl19*–injected mice at 24 h after stroke, followed by analysis of infarct volume. *n* = 10/group. **F** Neurological deficit scores of AAV-TIE2-siRNA-NC– and AAV-TIE2-siRNA-*Ccl19*–injected mice 24 h  after stroke. *n* = 10/group. Effects of *Ccl19* knockdown on behavioral recovery at different time points after stroke, as measured by the grid walking test (**G**), cylinder test (**H**), and adhesive removal test (**I**). *n* = 10/group. The data are presented as the means ± SEMs. **P < 0.01; ***P < 0.001; ^#^*P* < 0.05; ^##^*P* < 0.01; ^###^*P* < 0.001

We next investigated whether CCL19-CCR7 mediated the infiltration of Tsa cells into the brain parenchyma after stroke. Thus, AAV-TIE2-siRNA-*Ccl19* was intravenously injected into pretrained mice to specifically downregulate *Ccl19* in endothelial cells, which were then subjected to tMCAO on day 21 after AAV injection (Fig. 6A). We confirmed by flow cytometry that injection of AAV-TIE2-siRNA-*Ccl19* significantly reduced the expression of *Ccl19* in endothelial cells after 3 weeks (Fig. 6B). Next, we assessed the infiltration of Tsa cells from the ischemic brain hemisphere on day 1 after reperfusion and found that the injection of AAV-TIE2-siRNA-*Ccl19* significantly reduced the infiltration of Tsa cells into the brain parenchyma (Fig. 6C). Additionally, both the accumulation of the inflammatory factors TNF-α and IFN-γ in the peri-infarct area (Fig. 6D) and the infarct size (Fig. 6E) were significantly lower in the AAV-TIE2-siRNA-*Ccl19* group than in the AAV-TIE2-siRNA-NC group.

Subsequently, neurological deficit scores were assessed on day 1, and behavioral testing was performed on day 4–28. Compared with those in the AAV-TIE2-siRNA-NC group, mice treated with AAV-TIE2-siRNA-*Ccl19* exhibited significant improvements in neurological deficit scores (Fig. 6F) and improved performance, including a reduced number of foot faults in the grid-walking test on day 4, 7, and 14 after stroke (Fig. 6G). The cylinder test and adhesive removal test also revealed that, compared with the AAV-TIE2-siRNA-NC group, the AAV-TIE2-siRNA-*Ccl19* group exhibited a therapeutic benefit (Fig. 6H, I). These results suggest the therapeutic potential of treating ischemia‒reperfusion injury via direct intervention to reduce the chemotactic recruitment of CCR7-expressing Tsa cells.

### A CCR7 peptide designed to disrupt the CCL19‒CCR7 axis reduces ischemia‒reperfusion injury

**Fig. 7:**
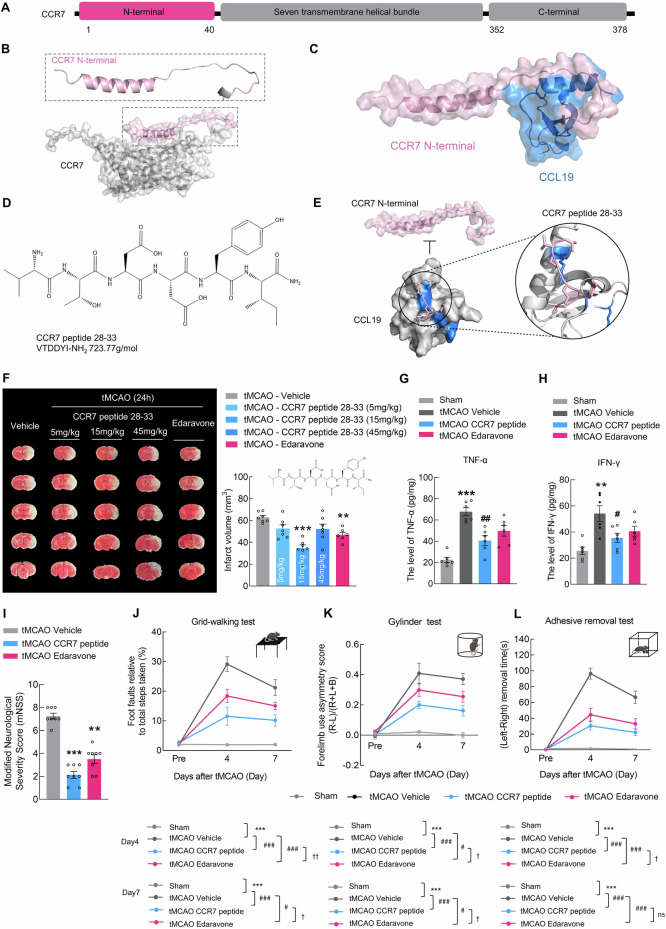
A CCR7 peptide designed to disrupt the CCL19‒CCR7 axis reduces IRI. **A** Schematic representation of CCR7. **B** Prediction of the structure of the CCR7 N-terminus via AlphaFold2. **C** Predicted binding conformation of the CCL19-CCR7 N-terminal heterodimer. **D** Potential peptide fragments of the CCR7 peptide 28–33. **E** Prediction of the binding site for the CCR7 peptide 28–33. **F** Representative TTC-stained sections 24 h after tMCAO in the vehicle, three different dosages of the CCR7 peptide 28–33 (5, 15, and 45 mg/kg), and edaravone groups, followed by an analysis of infarct volume. *n* = 7 animals in the sham group, three different dosages of CCR7 peptide 28-33 (5 mg/kg, 15mg/kg, and 45 mg/kg) group; n = 6 animals in the edaravone group. **G**, **H** The levels of the inflammatory factors TNF-α and IFN-γ in the peri-infarct area 24 h after tMCAO in the vehicle, CCR7 peptide 28–33 (15 mg/kg) and edaravone groups. *n* = 6/group. **I** Modified neurological severity scores of the vehicle, CCR7 peptide 28–33 (15 mg/kg), and edaravone groups at 24 h after stroke. *n* = 8/group. **J‒L** Functional recovery in the vehicle, CCR7 peptide 28–33 (15 mg/kg), and edaravone groups was assessed by the grid walking test (**J**), cylinder test (**K**), and adhesive removal test (**L**) at baseline and on day 4 and 7 after tMCAO. *n* = 7/group. The data are presented as the means ± SEMs. **P < 0.01; ***P < 0.001; ^#^*P* < 0.05; ^##^*P* < 0.01; ^###^*P* < 0.001, ^†^*P* < 0.05; ^††^*P* < 0.01

Finally, we designed a peptide targeting CCR7 to block the interaction between CCL19 and CCR7 (Fig. 7A–C), aiming to investigate its effect on Tsa cell infiltration into the brain parenchyma. Upon identifying potential peptide fragments, we screened two candidates suitable for experimental investigation: the CCR7 peptide 28–33 and the CCR7 peptide 29–35 (Figs. 7D and S19A, B). Binding site predictions for these peptides with CCR7 (Fig. 7E) suggested their potential to inhibit the interaction between CCR7 and CCL19. These two peptides were administered via tail vein injection at different dosages (5, 15, and 45 mg/kg) to tMCAO mice at 0.5 h after reperfusion. Compared with those in the vehicle group, the infarct volumes were significantly lower in the CCR7 peptide 28-33-treated group, with the maximal response at a dose of 15 mg/kg at 24 h after administration (Figs. 7F and S19C, D). However, CCR7 peptide 29–35 treatment did not significantly affect the infarct volume. Thus, 15 mg/kg CCR7 peptide 28–33 was selected for subsequent experimental interventions. Compared with vehicle treatment, CCR7 peptide 28–33 treatment substantially reduced the levels of TNF-α and IFN-γ, but not those of IL-6 or IL-1β, in the brains of the tMCAO model mice (Figs. 7G, H and S20A, B). Neurological deficit scores were significantly lower in CCR7 peptide 28-33-treated mice than in vehicle-treated mice at 24 h after reperfusion (Fig. 7I). Next, we investigated the effects of the CCR7 peptide 28–33 on sensorimotor function in mice. Compared with those in the vehicle group, the performance of the animals administered CCR7 peptides 28–33 improved, with fewer foot faults in the grid-walking test on day 4 and 7 after reperfusion (Fig. 7J). Similar results were recorded with the cylinder test, where animals injected with the CCR7 peptide 28–33 showed reduced bias on day 4 and 7 after PT stroke (Fig. 7K). The same therapeutic effect was found in the adhesive removal test, revealing significant differences between the CCR7 peptide 28–33 and vehicle groups (Fig. 7L). Collectively, these results showed that treatment with the CCR7 peptide 28–33 reduced neuroinflammation damage, alleviating behavioral dysfunction.

## Discussion

Our study delineates a tripartite mechanistic cascade underlying the pathogenic role of the brain-spleen-immune axis in cerebral ischemia‒reperfusion injury, integrating cerebral damage, sympathetic hyperactivity, and immune dysregulation. We established SDN as a neuroprotective intervention that disrupts stroke-induced catecholamine surges, reducing cerebral infarct volume while improving functional recovery in multiple behavioral paradigms. Through scRNA-seq analysis, we identified CD8^+^GZMK^+^CCR7^+^ Tsa cells as key immunopathological effectors whose circulating levels correlated significantly with neurological deficit scores and predicted long-term functional outcomes in AIS patients. Mechanistically, poststroke sympathetic hyperactivity drives Tsa cell mobilization via ADRB2-mediated β-adrenergic signaling, and endothelial-derived CCL19 orchestrates CNS infiltration through chemotactic CCR7 engagement. Therapeutically, we demonstrate two translationally relevant strategies: 1) SDN to disrupt sympathetic overactivation and 2) a CCR7-targeting peptide inhibitor to block Tsa cell infiltration. Our identification of Tsa cells as both biomarkers and therapeutic targets bridges the gap between experimental neuroimmunology and clinical stroke management, offering a paradigm for mechanistically informed immunomodulation in cerebrovascular diseases.

Neuroimmune transmission in the injured brain may establish an innovative treatment framework [37–39]. The spleen, as an early responder, serves as a crucial connection between brain injury and the peripheral immune system [40, 41]. Previous research has indicated that splenectomy may mitigate the development of cerebral immune responses, thereby improving the prognosis of stroke [42]. Consistent with the abovementioned findings, we demonstrated that activation of the splenic sympathetic nervous system drove the expansion of Tsa cells within the spleen, with subsequent influx in the peripheral blood and infiltration into the brain parenchyma. Thus, the splenic sympathetic nervous system could be a potential peripheral target for the treatment of ischemic stroke. Although the main finding of this study is the response of the spleen to brain injury, the mechanisms identified in the study may also involve CNS damage in other organs after stroke.

The temporal dynamics of T-cell infiltration play crucial roles in the progression of stroke pathology. Early infiltration of inflammatory T cells exacerbates inflammation and tissue damage in the brain [43]. However, T cells may have immunosuppressive effects that facilitate nerve repair, thereby improving stroke prognosis in later stages [44]. This dichotomy is attributed primarily to the diverse T-cell subtypes infiltrating the brain during different pathological phases poststroke. Among these, CD8^+^ T cells have garnered significant attention. Emerging evidence from stroke models has demonstrated that CD8^+^ T lymphocytes initiate CNS infiltration as early as 3 h following cerebral ischemia onset, preceding other adaptive immune cell populations in the neuroinflammatory cascade [45]. Interestingly, a specialized CD8^+^ T-cell subpopulation, CD8^+^CD122^+^CD49d^lo^ T regulatory-like cells (CD8^+^ TRLs), rapidly invades the injured brain and is essential for neuroprotection against acute ischemic brain injury [46]. This finding contrasts with the detrimental effects of general CD8^+^ T cells on ischemic brain injury [47]. Here, we observed significant enrichment of Tsa CD8^+^ T cells in the peri-infarct area as early as 6 h postischemia-reperfusion. This increase peaked at 24 h and remained stable for up to 72 h. Tsa cells exacerbate neuroinflammatory damage by secreting inflammatory factors, which play detrimental roles in cerebral protection during early stroke development and possibly in ischemia‒reperfusion injury progression. These observations underscore the complex and time-dependent roles of different T-cell subpopulations in stroke pathology, highlighting the need for targeted therapeutic approaches that consider the temporal dynamics of immune cell infiltration and activation.

Mounting evidence indicates that CD8^+^ T cells negatively impact stroke progression by inducing neuronal apoptosis [48, 49], exacerbating immune damage [50], and increasing the risk of intracerebral hemorrhage [16]. Upon stimulation, naive CD8^+^ T cells proliferate and differentiate into various memory and effector types. The memory CD8^+^ T-cell pool is notably heterogeneous in phenotype and function. GZMK is generally thought to be transiently expressed in memory CD8^+^ T cells and is downregulated as they differentiate into GZMB^+^ effector cells, which are characteristic of cytotoxic T lymphocytes (CTLs). Previous studies have indicated that GZMK^+^ CD8^+^ T cells play significant roles in various diseases. For example, age-associated GZMK-expressing CD8^+^ T (Taa) cells are a primary source of GZMK, enhancing inflammatory functions in nonimmune cells during aging [51]. GZMK^+^CXCR6^+^CD8^+^ T cells are tissue-resident memory T (Trm) precursors that promote IgA^+^ B-cell antibody secretion in the labial glands of patients with primary Sjögren’s syndrome [28]. In line with these findings, our study revealed that a subset of GZMK^+^ CD8^+^ T cells—Tsa cells, characterized by CX3CR1^−^CD27+CCR7^+^—infiltrated the brain parenchyma, increasing neuroinflammation during the early stage of ischemia‒reperfusion. The correlations between Tsa cells and the National Institutes of Health Stroke Scale (NIHSS) score and the Barthel index are crucial for understanding stroke outcomes and rehabilitation potential. Our results suggest that increased numbers of Tsa cells are associated with elevated NIHSS scores, indicating the potential role of these cells in amplifying neuroinflammatory processes and subsequent neurological deficits. Conversely, the negative correlation between Tsa cells and the BI suggests that increased inflammation may hinder the recovery of daily living activities, highlighting the complex interplay between the immune response and functional outcomes. These correlations emphasize the multifaceted impact of Tsa cells on stroke pathophysiology and recovery. This complexity underscores the need for a nuanced understanding of the role of specific immune cell subsets in stroke progression and resolution.

Chemokines have been established as key regulators of leukocyte infiltration [52]. Given that Tsa cells highly express CCR7, the CCR7 ligands CCL19 and CCL21 are likely pivotal in guiding the migration of Tsa cells. Referencing the Tabula sapiens Consortium [36] revealed distinct expression patterns of CCL19 and CCL21. In addition to being expressed in a specific subset of endothelial cells poststroke, as we discovered, CCL19 typically has low baseline expression predominantly in stromal cells under normal conditions. This finding highlights a specific cluster of endothelial cells that secrete CCL19, emphasizing their chemotactic role in recruiting CCR7^+^ pathological Tsa cells into the brain. In contrast, CCL21 is expressed mainly in endothelial cells, with higher levels observed in tissues or organs, including the trachea, muscle, vasculature, and lung. Our flow cytometry analysis of peripheral organs beyond the brain poststroke revealed Tsa cell accumulation in the lung but not in the heart or liver. The specific accumulation of Tsa cells in the lung suggests a potential mechanism for poststroke pulmonary complications, opening new avenues for investigating the multiorgan impact of stroke.

Our findings collectively redefine the neuroimmune axis in poststroke pathophysiology by establishing three interconnected paradigms: (1) Sympathetic hyperactivity drives early splenic catecholamine surges that license neurotoxic Tsa cell generation; (2) endothelial subtype-specific CCL19 production creates chemotactic gradients enabling CCR7-mediated CNS infiltration; and (3) this spleen-to-brain trafficking axis exhibits therapeutic vulnerability to both SDN and targeted CCR7 inhibition. These findings illuminate autonomic–immune crosstalk as a dynamic therapeutic target, compelling the initiation of clinical trials to evaluate SDN-adjuvant therapies and CCR7 antagonists in stroke rehabilitation protocols. By elucidating the dynamics of neuroimmune injury cascades, our study provides both mechanistic insights and actionable targets for interrupting the self-perpetuating inflammation that impedes poststroke recovery.

## Materials and methods

### Human studies

All participants, including patients and healthy volunteers, were enrolled from the Affiliated Hospital of Xuzhou Medical University. Ethical approval for the study was obtained from the Clinical Research Ethics Committee of the Affiliated Hospital of Xuzhou Medical University (ID: XYFY2023-KL239-01). The research adhered to the International Ethical Guidelines for studies involving human subjects, as stated in the Helsinki Declaration. Informed consent was obtained from all the subjects at the time of enrollment. Postmortem brain samples were obtained from the Chinese Brain Bank Center (CBBC) (Wuhan, China) (http://www.cbbcnet.cn/). The experiments were approved by the South-Central University for Nationalities Research Ethics and Safety Committee (Approval No. 2021-scuec-034).

### Animals

Adult male C57BL/6 mice (6–8 weeks old) were purchased from Gem Pharmatech Co., Ltd. (Nanjing, China). All animal experiments were approved by the Institutional Animal Care and Use Committee (IACUC) at the Medical School of Southeast University (approval ID 20220225014) and performed in accordance with the Animal Research: Reporting of In Vivo Experiments (ARRIVE) guidelines. The animals were housed under conditions of constant temperature (approximately 21 °C) and humidity (~50%) and maintained on a 14-h light/10-h dark cycle with lights on at 7:00 a.m. A mouse diet (SFS9112, Xietong Shengwu) and water were provided ad libitum. The sample size required for animal research was based on previous experimental results and was similar to the sample size commonly used in the field. The mice were coded and randomly divided into experimental and control groups.

### Human blood samples

Blood samples for single-cell RNA sequencing were collected from 3 AIS patients (2 males and 1 female) and 3 healthy controls (2 males and 1 female). The available clinical characteristics of these three patients are summarized in Table S1. Blood samples for flow cytometry were collected from 60 patients with AIS (47 males and 13 females) and 60 healthy individuals (43 males and 17 females). There was no significant difference in the age of the recruited subjects (AIS vs. control: 65.88 ± 1.49 vs. 66.68 ± 1.22 years, *P* = 0.150). The available clinical characteristics of these participants are summarized in Table S2. Ischemic stroke was diagnosed on the basis of clinical presentation in conjunction with a diffusion-weighted imaging (DWI) lesion on magnetic resonance imaging (MRI) or a new lesion on a delayed computed tomography (CT) scan. For all participants involved in our research, we excluded stroke and nonstroke patients with active malignant disease or neurologic and psychiatric disorders, patients who had undergone surgery within the last 3 months, and patients who had taken low-molecular-weight heparin or nonfiltered heparin medications within the last month. Outcomes were assessed by telephone 3 months after stroke onset.

### Postmortem brain samples

Frozen frontal cortex samples were dissected from Brodmann area 4 or 6. The brains were dissected by trained neuroanatomists and stored at –80 °C. The cause of death for each AIS individual was ischemic stroke, whereas nonstroke controls were individuals who died suddenly and did not have evidence of stroke or neurological or psychiatric diseases (Table S3).

### Single-cell collection

Peripheral blood mononuclear cells (PBMCs) were obtained from whole blood samples via density gradient centrifugation. Briefly, after the plasma was collected by centrifugation, whole blood was diluted 1:1 with 1× PBS, layered on top of Ficoll-Paque Plus (GE Healthcare, 17-1440-02), and centrifuged at 500 × *g* for 30 min. The cells were washed twice and lysed with ACK lysis buffer (C3702, Beyotime Biotech, China) for 5 min at 4 °C to completely remove red blood cells. The cell suspensions were filtered through a 70-μm cell strainer. A cell viability > 90% was required for the subsequent construction of libraries. All the samples were subsequently stored at −80 °C overnight and then transferred to liquid nitrogen for long-term storage.

### Single-cell library generation and sequencing

Single-cell libraries were created via the Chromium Single-cell 3’ library preparation kit user guide (10x Genomics). All libraries generated for this work were sequenced via a NovaSeq 6000 system (Illumina) with 150 bp paired-end sequencing, and each library was sequenced for the whole lane. To generate single-cell gel beads-in-emulsions (GEMs), a cellular suspension, barcoded single-cell 3’ gel beads, and partitioning oil were loaded onto a Chromium Chip A. GEM reverse transcription (GEM-RT) was carried out in a Veriti 96-well thermal cycler (Thermo Fisher Scientific). Silane magnetic beads were used to remove residual biological reagents and primers from the mixture after the GEM reaction. PCR amplification of the full-length barcode cDNA was then performed to generate the quantities required for library assembly. Indexed sequencing libraries were generated via the Chromium Single-cell 3’ Library Construction Kit according to the instructions. A real-time PCR system (Life Technologies, USA) was used to quantify the barcoded sequencing libraries.

### Preprocessing of single-cell RNA-seq data

Unique molecular identifier (UMI) count matrices and associated cellular barcodes were obtained for each gene by matching reads against the human transcriptome (GRCh38) via Cell Ranger software (v3.1.0, 10x Genomics). To ensure data quality, we retained only genes expressed in at least three cells and in cells with 400 or more genes. Cells that did not meet the quality criteria were excluded on the basis of the following conditions: (1) the number of expressed genes was less than 500 or greater than 4000; (2) the number of UMIs was less than 1000 or greater than 15,000 per cell; and (3) more than 8% of the UMIs were mapped to mitochondrial genes. After deleting unnecessary cells from the dataset, we employed a global-scaling normalization method, “LogNormalize,” that normalizes the gene expression measurements for each cell by the total expression, multiplies this value by a scale factor (10,000 by default), and log-transforms the results. The formula was as follows: A gene expression level = log (1 + (UMIA ÷ UMITotal) × 10000).

### Visualization and cell identification

To visualize the data, principal component analysis (PCA) was used to further reduce the dimension of the scaled integrated data matrix to project the unit into a two-dimensional space. On the basis of the classification of cell subpopulations, single-cell classification was visualized via uniform manifold approximation and projection (UMAP). Cluster identity was assigned by finding differentially expressed genes and then comparing these cluster-specific genes with previously published datasets.

To investigate specific marker genes for each subpopulation, we looked for genes that were upregulated in each cluster. Seurat’s Wilcoxon rank sum test was used to investigate gene expression differences across cell clusters. Important upregulated genes were identified by a series of criteria. First, the percentage of cells expressing the genes was greater than 25% of all cells in a given cluster. Second, the log fold-change of the average expression (logFC) ≥ 0.360674; that is, gene upregulation multiple ≥1.28. Third, *P* ≤ 0.05 was considered significant. Heatmaps or violin plots were then used to depict the distribution of marker genes. The identities of clusters of cells were manually annotated via known marker genes in published articles with the help of the R package *SingleR* (v1.0.1). The markers used for T-cell identification are presented in Table S4.

### GO enrichment analysis

GO enrichment analysis identified all GO terms that were significantly enriched in DEGs compared with the genomic background. It also looks for differentially expressed genes that correspond to biological functions. First, all peak-related genes were mapped to GO terms in the Gene Ontology database (http://www.geneontology.org/), gene counts were determined for each term, and hypergeometric tests were used to identify highly enriched GO terms in differentially expressed genes compared with the genome background.

### Transient middle cerebral artery occlusion (tMCAO)

tMCAO was performed according to a previous study [53–56]. Briefly, anesthesia was induced with 3% isoflurane, and the anesthesia was maintained with 1.5% isoflurane in 30% oxygen and 70% nitrous oxide using a face mask. The right external carotid artery was exposed, and a silicone rubber-coated 6-0 nylon filament (602356PK5Re, Doccol, USA) was inserted and advanced 9–10 mm to the carotid bifurcation along the internal carotid artery (ICA) and to the origin of the middle cerebral artery (MCA). One hour after occlusion, the filament was removed to restore blood flow to the middle cerebral artery territory. A temperature-controlled heating pad was used to maintain the temperature at 37.0 ± 0.5 °C during surgery and the recovery period.

### Splenic denervation

In a previous study, splenic denervation was performed by treating the splenic nerve plexuses with anhydrous ethanol before they entered the spleen along the vasculature. After anesthesia with 20 mg/kg sodium pentobarbital, the peritoneal cavity of each mouse was accessed through a midline abdominal incision. The spleen was isolated away from the peritoneal cavity with forceps so that the three main supplying vasculature trees were clearly exposed. With the peritoneal cavity and other organs protected with a moistened cotton pad, under a dissection microscope, the three main supplying vasculature trees were fully exposed to anhydrous ethanol-soaked cotton tips for 5–10 s at 5 s intervals seven times in total to deplete the splenic nerve. Care was taken to avoid excessive ethanol dripping from the cotton tip and to avoid visible vessel spasms, which could lead to permanent damage to blood vessels and cause splenic necrosis and complete organ absorption. For the sham-operated mice, saline (pH 7.4) instead of anhydrous ethanol was repeatedly used with identical surgical methods. The mice were allowed to recover for two weeks before the other experiments.

### Flow cytometry

The samples were prepared as previously described and subsequently resuspended in 2% fetal bovine serum in 1× PBS. The samples were blocked with FcR Blocking Reagent (130-092-575, Miltenyi Biotec) for 15 min and then incubated with surface antibodies and a live/dead cell marker for 30 min at 4 °C in the dark. The cells were subsequently fixed and permeabilized with a Transcription Factor Buffer Set (562574, BD Pharmingen) or Fixation/Permeablization Kit (554714, BD Pharmingen) according to the manufacturer’s instructions and then stained with antibodies for 30 min at 4 °C. Finally, the cells were washed and resuspended in ice-cold FAC buffer for analysis via a flow cytometer.

### Fluorescence-activated cell sorting (FACS)

The brain tissues of the mice were collected and then temporarily placed on ice. The tissues were dissociated and digested at 37 °C for 1 h with papain (2 mg/ml, LS003119, Worthington) in RPMI 1640 medium (C11875500BT, Gibco). Dispersed cells were passed through a nylon mesh (70 μm) and collected by centrifugation. The cells were subsequently resuspended in a 30% Percoll density gradient (17-0891-09, GE Healthcare) and centrifuged for 25 min (900 × g, 25 °C). The cells were isolated by collection of the bottom fraction in 30% Percoll. The samples were blocked with FcR Blocking Reagent (130-092-575, Miltenyi Biotec) and resuspended in PBS containing 2% FBS. Astrocytes, microglia, neurons, and endothelial cells were assayed for surface antigens. The cells were stained with PE-conjugated anti-mouse ACSA-2 (130-116-244, Miltenyi Biotec), FITC-conjugated anti-mouse/human CD11b (101205, BioLegend), PerCp-Cy5.5-conjugated anti-mouse CD45 (561869, BD Pharmingen), APC-conjugated anti-mouse NCAM-1/CD56 allophycocyanin MAb (FAB7820A-100, R&D), and Brilliant Violet 605™-conjugated anti-mouse CD31 (102427, BioLegend) antibodies. After staining, the samples were sorted with a FACSAria II SORP (BD Biosciences, USA), and the data were analyzed via FlowJo_V10 software. The samples were gated for ACSA-2^+^ (astrocytes), ACSA-2^-^CD11b^+^CD45^dim^ (microglia), NCAM-1/CD56^+^ (neurons), and CD31^+^ (endothelial cells). An RNeasy®-Micro Kit (74004, QIAGEN) was used for RNA extraction.

### Quantitative real-time polymerase chain reaction (qPCR)

qPCR was performed according to our previous study in an Applied Biosystems qPCR System [57]. Total RNA was extracted via TRIzol reagent (15596026CN, Invitrogen). cDNA was acquired via the HiScript QRT SuperMix for qPCR Kit (R123-01, Vazyme). The mRNAs were quantified via SYBR Green qPCR Master Mix (Q141-02, Vazyme) and calibrated to the internal control β-actin. The cycle threshold was detected with a StepOne^TM^ Real-Time PCR instrument (StepOne^TM^ 4480845, Applied Biosystems^®^). The primers were synthesized by Invitrogen and are listed in the *Key Resources Table*.

### Western blot analysis

Proteins were extracted in RIPA lysis buffer (P0013B, Beyotime), separated on sodium dodecyl sulfate polyacrylamide gels (8% and 12%) and electrophoretically transferred onto polyvinylidene fluoride membranes according to previously described methods [58]. After incubation with blocking buffer, the membrane was incubated with antibodies against CCL19 (1:1000, DF2909, Affinity Biosciences), tyrosine hydroxylase (TH) (1:1000, AB152, Millipore), or beta-actin (1:3000, 66009-1-lg, Proteintech) overnight at 4 °C. The membranes were then incubated with an HRP-conjugated affinipure goat anti-mouse or rabbit IgG (H + L) secondary antibody (1:2000, SA00001-1 or SA00001-2, Proteintech). The signals were detected via an automatic chemiluminescence image analysis system (Tanon 5200, Tanon Science & Technology). The individual protein bands were quantified via densitometry via ImageJ software.

### In vivo MRI scanning

In vivo MRI was performed via a 7.0 Tesla small animal MRI scanner (Bruker, Ettlingen, Germany; PharmaScan). The mice were anesthetized with 2% isoflurane delivered through a nose cone, and their respiratory rate and body temperature were monitored via a physiology monitor. Twelve axial slices with a slice thickness of 1 mm, a matrix of 256 × 256, and a field of view of 20 × 20 mm were positioned over the brain, excluding the olfactory bulb. The total scan time was 5 min. The percentage of infarct volume was calculated from T2-weighted images. Briefly, the percentage of infarct volume is presented as the lesion volume/the contralateral hemisphere volume covering the whole slices of T2-weighted images. The lesion volume was acquired on the basis of the high-signal area of the T2-weighted images from which the artifact of brain edema was subtracted. The infarct volume was traced and calculated via ImageJ software (NIH).

### 2,3,5-Triphenyltetrazolium chloride (TTC) staining

The infarct volume was evaluated 24 h after tMCAO. The mice were anesthetized with 1% pentobarbital sodium and perfused with 0.01 M PBS. Following perfusion, the mice were decapitated, and their brains were swiftly collected and frozen at −20 °C for 6 min. The brains were then coronally sectioned into 1-mm-thick slices on ice using a brain matrix. The brain slices were subsequently incubated in 1% TTC (Sigma‒Aldrich, T8877) at 37 °C for 10 min and fixed in 4% paraformaldehyde (PFA) to visualize and quantify the size and extent of the infarction. To account for brain swelling, the infarct area was determined by subtracting the area of noninfarcted tissue in the ipsilateral hemisphere from that of the intact contralateral hemisphere. The infarct volume was then calculated by integrating the infarct areas across all slices of each brain [59].

### Measurements of neurological deficit

The neurological deficit test was performed by an experimenter blinded to the experimental groups 24 h after tMCAO. The evaluation of neurological function was conducted via the modified neurological severity score (mNSS) test [60]. The score was graded on a scale of 0–14 (normal score, 0; maximum score, 14). One point is awarded for the inability to perform a test or for the lack of a tested reflex. A score of 10 to 14 indicates severe injury; a score of 5 to 9 indicates moderate injury; and a score of 1–4 indicates mild injury.

### Mouse behavioral tests

Mouse behavioral tests were performed by an independent investigator who was blinded to the experimental groups, and the data were analyzed by another investigator.

For the grid-walking task, an elevated grid area of 32 cm × 20 cm × 50 cm (length × width × height) made of 12 mm square wire mesh was used [61, 62]. Each mouse was placed individually on the wire grid and allowed to freely move until a minimum of 100 steps were taken by the left forelimb. A camera was positioned beneath the grid to record stepping errors (foot faults). In brief, it was evaluated as a foot fault: (1) if a step was not able to provide support and the foot went through the grid hole; (2) if the mouse was resting while the grid was at the level of its wrist. The numbers of foot faults and nonfaults for each limb were counted. The ratio was calculated as follows: number of foot faults/(number of foot faults + number of nonfaults) × 100%. Differences in the degree of locomotion of the animals were excluded by calculating the ratio of foot faults to total steps taken.

For the cylinder test [63], the mice were placed inside a plastic cylinder (15 cm tall with a diameter of 10 cm) and videotaped for 5 min. The score was calculated as follows: (number of right hands − number of left hands)/(number of right hands + number of left hands + number of both hands).

For the adhesive removal somatosensory test [55, 64], small pieces of adhesive-backed paper dots (of equal size, 25 mm^2^) were used as bilateral tactile stimuli occupying the distal-radial region on the wrist of each forelimb. The time for the mice to remove each stimulus from the forelimb was recorded for three trials per day. Individual trials were separated by at least 5 min. Before surgery, the animals were trained for 3 days. Once the dots were removed within 10 s, the mice were subjected to stroke. The result was calculated as follows (time in seconds): time of left hand time of right hand.

### Immunofluorescence staining

Before staining, the brain tissues were cut into 30-μm coronal slices, subsequently incubated with 0.3% Triton X-100 (Aladdin, T109027, in PBS) for 15 min and blocked with 10% goat serum (ZLI-9056, ZSGB-BIO, in 0.3% Triton X-100) for 60 min at room temperature. Next, the sections were incubated with a GZMK antibody (PA5-100743, Invitrogen) or CD3 antibody (MCA1477, Bio-Rad) at 4 °C overnight. On the following day, the slices were washed with PBS and incubated with Alexa Fluor 488-conjugated goat anti-rat IgG (A48262, Invitrogen) or Alexa Fluor 594-conjugated goat anti-rabbit IgG (A11012, Invitrogen) for 1 h at room temperature. After a final washing step with PBS, the sections were mounted onto glass slides. Images were captured via a confocal microscope (Zeiss, LSM 800).

### Enzyme-linked immunosorbent assay (ELISA)

The levels of cytokines (TNF-α, IFN-γ, IL-1β and IL-6) in the peri-infarct tissue extracts were analyzed via commercially available ELISA kits according to the manufacturer’s instructions. ELISA kits for mouse TNF-α (LA128801H), IFN-γ (LA128805H), IL-6 (LA128802H), and IL-1β (LA128804H) were purchased from Lapuda Biological Technology Co. Ltd.

### Peptide synthesis

Peptides were synthesized via Fmoc-based solid-phase synthesis on Rink amide MBHA resin (0.34 mmol/g loading). The resin (1 mmol) was swelled with dichloromethane (DCM) and then deprotected (10 min × 2) using a Fmoc deprotection solvent (N,N-dimethylformamide, DMF, containing 20% piperidine and 0.1 mol/l pure Oxyma) at 30 °C. The peptides were washed with DMF and DCM (1 min × 3). A solution of Fmoc-protected amino acid (1 mmol), N,N’-diisopropylcarbodiimide (1 mmol), or pure Oxyma (1 mmol) in N-methylpyrrolidone was added, and the mixture was allowed to react with the resin at 60 °C for 20 min. After the reaction, the resin was washed with DMF and DCM and deprotected for the next amino acid coupling. The peptide was cleaved from the resin using a cocktail of trifluoroacetic acid (TFA), triisopropylsilane, and H_2_O (95:2.5:2.5, V/V) for 2 h at RT and concentrated under a stream of nitrogen. The crude peptides were obtained by precipitation with 30 ml of ice-cold tert-butyl methyl ether, centrifugation at 3500 rpm for 5 min, purification by reversed-phase high-performance liquid chromatography (RP-HPLC) and analysis via mass spectrometry (MS).

### Statistics

All the experiments presented in this study were repeated at least three times. Significance was assessed with Student’s *t* test (2-tailed) for comparisons of 2 groups or the Mann‒Whitney *U* test for nonnormally distributed data. One-way analysis of variance (ANOVA) followed by Tukey’s post hoc test was used to compare data from three or more groups with one variable. For comparisons of two or more variables among multiple groups, two-way ANOVA followed by the Holm–Sidak post hoc test was used. *P* < 0.05 was considered to indicate statistical significance. The data were analyzed with GraphPad Prism 8.0. All the data are presented as the means ± SEMs.

## Supplementary information

Supplemental Materials

Table S4. T sebset markers

Supplementary information-Original western blot images

## Data Availability

The single-cell RNA sequencing data generated during this study are available in the Genome Sequence Archive (GSA) database with public access (CRA016385, HRA007397).
