# Supplementary material for: CD8+GZMK+CD27+CCR7+ T cells mobilized by splenic sympathetic nerves aggravate brain ischemia‒reperfusion injury via CCL19-positive endothelial cells
Source: Cell Mol Immunol. 2025 Jul 14;22(9):1061–76. doi: 10.1038/s41423-025-01311-9 (PMC12398583; doi:10.1038/s41423-025-01311-9)
Supplement: Supplementary file 1 — Supplemental Materials [file 41423_2025_1311_MOESM1_ESM.pdf]

SUPPLEMENTAL MATERIALS

CD8⁺GZMK⁺CD27⁺CCR7⁺ T cells mobilized by splenic sympathetic nerve aggravate brain ischemia-reperfusion injury via CCL19-positive endothelial cells

Ying Bai[1†], Hui Ren[1†], Shuo Leng[2†], Mengqin Yuan[3], YiXin Jiang[4], Shenyang Zhang[1, 5], Yu Wang[1], Minzi Ju[1], Zhi Wang[2], Wen Xi[1], Lian Xu[1], Bingjing Zheng[1], Daxing Li[1], Xinchen Huo[1], Tianhao Zhu[1], Beicheng Zhang[1], Ling Shen[1], Yuan Zhang[1], Wei Jiang[6], John H. Zhang[7], Bing Han[1, 11*], Honghong Yao[1, 8, 9, 10 *]

[1] Department of Pharmacology, Jiangsu Provincial Key Laboratory of Critical Care Medicine, School of Medicine, Southeast University, Nanjing 210009, China;

[2] Center of Interventional Radiology and Vascular Surgery, Department of Radiology, Zhongda Hospital, Medical School, Southeast University, Nanjing 210009, China;

[3] Department of Biomedical Engineering, Nanjing University of Aeronautics and Astronautics, Nanjing 211106, China;

[4] Shanghai Frontiers Science Center of TCM Chemical Biology, Institute of Interdisciplinary Integrative Medicine Research, Shanghai University of Traditional Chinese Medicine, Shanghai 201203, China;

[5] Department of Neurology, Affiliated Hospital of Xuzhou Medical University, Xuzhou 221006, China;

[6] Fujian Key Laboratory of Precision Medicine for Cancer, the First Affiliated Hospital, Fujian Medical University, Fuzhou 350005, China;

[7] Department of Physiology, School of Medicine, Loma Linda University, Loma Linda CA, 92350, USA;

[8] Co-innovation Center of Neuroregeneration, Nantong University, Nantong 226001, China;

[9] Institute of Life Sciences, Key Laboratory of Developmental Genes and Human Disease, Southeast University, Nanjing 210096, China;

[10] Center for Global Health, School of Public Health, Nanjing Medical University, Nanjing 211166, China.

[11] Nanjing Pukou People's Hospital, Liangjiang Hospital, Southeast University, Nanjing 211899, China.

*Correspondence: yaohh@seu.edu.cn (H.Y), hanb@seu.edu.cn (B.H).

[†] Ying Bai, Hui Ren, Shuo Leng contributed equally to this work.

Key resource table

REAGENT or RESOURCE	SOURCE	IDEN TIFIER
Antibodies		
Fixable Viability Stain 780	BD Pharmingen	Cat# 565388
BV786·Mouse Anti-Human.CD45 (HI30)	BD Pharmingen	Cat# 563716
BV421 Mouse Anti-Human CD19 (HIB19)	BD Pharmingen	Cat# 562440
BV510 Mouse Anti-Human CD56 (NCAM16.2)	BD Pharmingen	Cat# 563041
PE Mouse Anti-Human HLA-DR (G46-6)	BD Pharmingen	Cat# 555812
FITC Mouse Anti-Human CD3 (UCHT1)	BD Pharmingen	Cat# 555332
R718 Mouse Anti-Human CD4 (M-T477)	BD Pharmingen	Cat# 752165
PerCP-Cy5.5 Mouse Anti-Human CD8 (SK1)	BD Pharmingen	Cat# 565310
BV650 Mouse Anti-Ki-67 (B56)	BD Pharmingen	Cat# 563757
BV650 Mouse Anti-Human CD45 (HI30)	BD Pharmingen	Cat# 563717
FITC Mouse Anti-Human CD3 (OKT3)	BD Pharmingen	Cat# 566783
BV786 Mouse Anti-Human CD45RA (HI100)	BD Pharmingen	Cat# 563870
BV421 Mouse Anti-Human CD197(CCR7) (150503)	BD Pharmingen	Cat# 562555
BV605 Mouse Anti-Human CD25(2A3)	BD Pharmingen	Cat# 562660
PE-Cy7 Mouse Anti-Human CD127(HIL-7R-M21)	BD Pharmingen	Cat# 560822
Alexa Fluor 647 Mouse Anti-Human Granzyme K(G3H69)	BD Pharmingen	Cat# 566655
BV510 Mouse Anti-Human Granzyme B(GB11)	BD Pharmingen	Cat# 563388
FITC Mouse Anti-Human CD45	BD Pharmingen	Cat# 555482
BV510 Mouse Anti-Human CD3 (UCHT1)	BD Pharmingen	Cat# 563109
BUV395 Mouse Anti-Human CD8(RPA-T8)	BD Pharmingen	Cat# 563795
PE Mouse Anti-Human CD27 (M-T271)	BD Pharmingen	Cat# 555441
BV421 Rat Anti-Human CX3CR1 (2A9-1)	BD Pharmingen	Cat# 565800
BB700 Rat Anti-Human CCR7 (CD197) (3D12)	BD Pharmingen	Cat# 566437
BV786 Mouse Anti-Human IFN-γ (4S. B3)	BD Pharmingen	Cat# 563731
BV650 Mouse Anti-Human TNF (MAb11)	BD Pharmingen	Cat# 563418
BUV395 Rat Anti-Mouse CD45 (30-F11)	BD Pharmingen	Cat# 564279
BV510 Hamster Anti-Mouse CD3e (145-2C11)	BD Pharmingen	Cat# 563024
PerCP-Cy5.5 Rat Anti-Mouse CD8a (53-6.7)	BD Pharmingen	Cat# 551162
Ms CD62L PE MEL-14	BD Pharmingen	Cat# 553151
MS CD44 BV650 IM7	BD Pharmingen	Cat# 740455
PE-Cy7 Hamster Anti-Mouse CD27(LG.3A10)	BD Pharmingen	Cat# 563604
BV421 Rat Anti-Mouse CD197 (CCR7) (4B12)	BD Pharmingen	Cat# 562675
Alexa Fluor 647 Rat Anti-Mouse CX3CR1(Z8-50)	BD Pharmingen	Cat# 567805
Granzyme K antibody (FITC)	biorbyt	Cat# orb102688
PE anti-mouse ACSA-2	Miltenyi Biotec	Cat# 130-116-244
FITC anti-mouse/human CD11b	BioLegend	Cat# 101205
PerCP-Cy5.5 Rat Anti-Mouse CD45 (30-F11)	BD Pharmingen	Cat# 561869

APC anti-mouse NCAM-1/CD56	R&D Systems	Cat# FAB7820A
Brilliant Violet 605™ anti-mouse CD31	BioLegend	Cat# 102427
Granzyme K Polyclonal Antibody	Invitrogen	Cat# PA5-100743
Rat anti Human CD3	BIO-RAD	Cat# MCA1477
Goat anti-Rat IgG (H+L) Highly Cross-Adsorbed Secondary Antibody, Alexa Fluor™ Plus 488	Invitrogen	Cat# A48262
Goat anti-Rabbit IgG (H+L) Cross-Adsorbed Secondary Antibody, Alexa Fluor™ 594	Invitrogen	Cat# A-11012
Anti-Tyrosine Hydroxylase (TH)	Millipore	Cat#AB152
CCL19 Antibody	Affinity Biosciences	Cat# DF2909
β-Actin Antibody	Proteintech	Cat# 66009-1-lg
Goat anti-mouse IgG secondary antibody	Proteintech	Cat# SA00001-1
Goat anti-rabbit IgG secondary antibody	Proteintech	Cat# SA00001-2
Bacterial and virus strains		
AAV-TIE2-siRNA-CCL19	GeneChem Co., Ltd	N/A
AAV-TIE2-siRNA-NC	GeneChem Co., Ltd	N/A
Biological samples		
PBMCs	Affiliated Hospital of Xuzhou Medical University	N/A
Chemicals, peptides, and recombinant proteins		
Ficoll-Paque PLUS	GE Healthcare	Cat# 17-1440-02
ACK lysis buffer	Beyotime Biotech, China	Cat# C3702
Transcription Factor Buffer Set	BD Pharmingen	Cat# 562574
Fixation/Permeablization Kit	BD Pharmingen	Cat# 554714
Papain	Worthington	Cat# LS003119
RPMI 1640 medium	Gibico	Cat# C11875500BT
Percoll	GE Healthcare	Cat# 17-0891-09

FcR Blocking Reagent	Miltenyi Biotec	Cat# 130-092-575
RNeasy®-Micro Kit	QIAGEN	Cat# 74004
TRIzol	Invitrogen	Cat# 15596026CN
HiScript Q RT SuperMix for qPCR	Vazyme	Cat# R123-01
SYBR Green qPCR Master Mix	Vazyme	Cat# Q141-02
RIPA lysis buffer	Beyotime	Cat# P0013B
2,3,5-Triphenyltetrazolium chloride (TTC)	Sigma-Aldrich	Cat# T8877
Triton X-100	Aladdin	Cat# T109027
DAPI	Southern Biotech	Cat# 0100-20
Critical commercial assays		
Mouse TNF-α	Lapuda Biological Technology Co. Ltd	Cat# LA128801H
Mouse IFN-γ	Lapuda Biological Technology Co. Ltd	Cat# LA128805H
Mouse IL-6	Lapuda Biological Technology Co. Ltd	Cat# LA128802H
Mouse IL-1β	Lapuda Biological Technology Co. Ltd	Cat# LA128804H
Experimental models: Organisms/strains		
Mouse: C57BL/6	Gem Pharmatech Co, Ltd	Cat# N000013
Software and algorithms		
Fiji	Image J	https://imagej.net/software/fiji/
FlowJo™ v10	Tree Star	https://www.flowjo.com/solutions/flowjo/downloads

Prism 8.0.2	GraphPad	https://www.graphpad.com/scientific-software/prism/
Graphical design software	BioRender	https://biorender.com/

Table S1. Demographic and clinical characteristics of stroke and non-stroke samples

	Non-stroke	AIS	Statistical analysis(*P*)
**Demographic characteristics**			
Total, n	60	60	1.000
Age, mean (SEM), years	66.68(1.22)	65.88(1.49)	0.150
Female, n (%)	17(28.33)	13(21.67)	0.399
Vascular risk factors, n (%)			
Hypertension	20(33.33)	31(51.67)	0.042
Smoke	6(10.00)	18(30.00)	0.006
Hypercholesterolemia	6(10.00)	9(15.00)	0.255
Diabetes mellitus	9(15.00)	19(31.67)	0.031
Previous TIA/stroke/MI	5(8.33)	10(16.67)	0.168
**Laboratory parameters, mean (SEM)**			
Glucose (mmol/L)	5.54(0.20)	6.38(0.26)	0.034
AST (IU/L)	19.98(1.40)	24.05(0.91)	< 0.001
ALT (IU/L)	18.93(1.48)	21.55(1.32)	0.014
γ-GT (IU/L)	22.75(1.35)	35.33(5.18)	0.245
Total cholesterol (mmol/L)	4.06(0.14)	4.27(0.16)	0.578
Triglycerides (mmol/L)	1.45(0.13)	1.42(0.10)	0.585
HDL (mmol/L)	1.04(0.04)	1.03(0.03)	0.161
LDL (mmol/L)	2.36(0.11)	2.54(0.13)	0.053
WBC (10^9/L)	5.97(0.16)	8.57(0.38)	< 0.001
RBC (10^{12}/L)	4.65(0.04)	4.79(0.07)	0.001
Hb(g/L)	141.05(1.50)	146.77(2.29)	0.006
PLT (10^9/L)	219.95(5.63)	222.38(12.48)	0.362
BUN (mmol/L)	6.16(0.23)	6.14(0.24)	0.831
Cr (μmol/L)	63.25(1.73)	68.35(2.75)	0.265
Total protein (g/L)	67.30(0.68)	70.79(0.91)	0.003
Albumin	41.24(0.44)	41.37(0.53)	0.203
Lp (a) (g/L)	259.52(31.77)	255.57(29.06)	0.846
INR	0.93(0.02)	0.93(0.01)	0.218
Prothrombin time (s)	10.76(0.16)	10.73(0.09)	0.266
APTT (s)	27.72(0.48)	26.02(0.34)	0.053
FIB (g/L)	2.62(0.08)	2.71(0.10)	0.684

Table S2. Summary of patients treated with mechanical thrombectomy based on the rapid processing of perfusion and diffusion

Patient	Age/Sex	Imaging	Infarct core	mismatch	Time from onset (h)	NIHSS	NIHSS at Day 1	NIHSS at Day 7	mRS at 3 months	Matched Non-stroke Control
**1**	66/Male	Left MCA occlusion	38ml	Mismatch volume168 ml, ratio 4.4	3.5	10	6	4	1	69/Male
**2**	69/Male	Right MCA occlusion	13ml	Mismatch volume72 ml, ratio 6.5	3.5	11	11	8	3	65/Male
**3**	72/Female	Right MCA occlusion	-	Mismatch volume74 ml, ratio infinite	2.5	12	4	0	0	72/Female

Table S3. Information about postmortem brain samples

	Non-stroke control (n = 6)	AIS patients (n = 6)	P value
**Age (years)**	62.33±1.05	62.83±2.39	0.851
**Gender (male/female)**	5/1	5/1	NA
**PMI (h)**	10.8±1.02	9.05±0.30	0.133
**Brain weight (g)**	1300±61.99	1203±42.38	0.226
**Storage time (years)**	1.65±0.40	2.125±0.17	0.304
**Clinical diagnoses**	Toxemia (2)	AIS (6)	NA
	Pneumonia (1)		
	Respiratory failure (1)		
	Hypertension (2)		

Note: AIS, acute ischemic stroke; PMI, post-mortem interval; NA, not applicable.

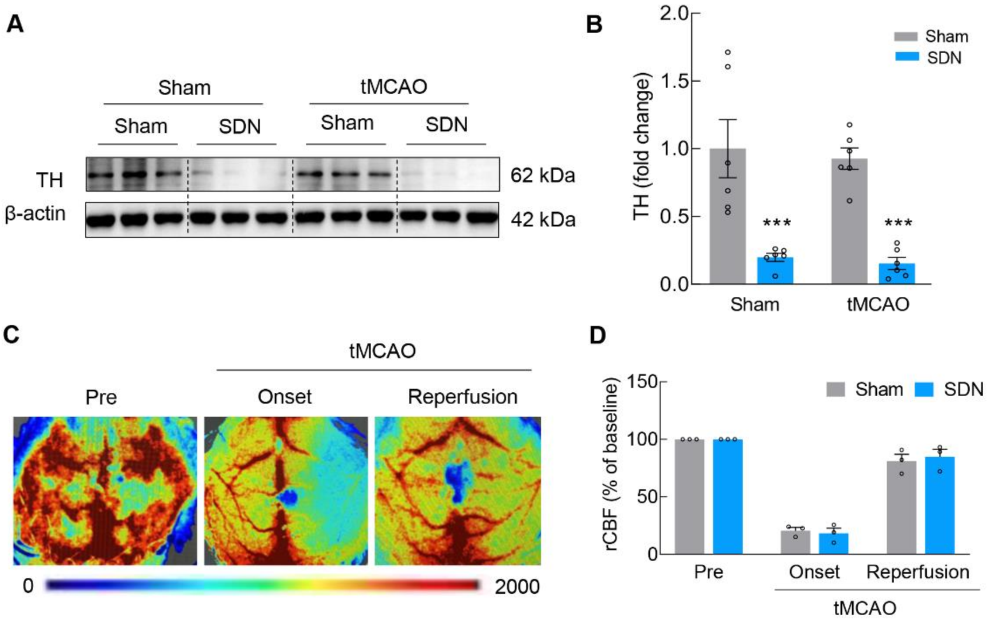

Supplementary Figure 1. Evaluation of splenic sympathetic denervation efficiency and representative cerebral blood flow images in tMCAO mice. A-B. Western blot analysis of Tyrosine Hydroxylase (TH) after SDN in tMCAO mice. Mice were treated with tMCAO on day 7 after SDN. The expression of TH was determined on day 7 after SDN. n = 6/group. **C-D.** Representative cerebral blood flow images at three timepoints (Pre, Onset, and Reperfusion) in tMCAO mice, followed by analysis of regional cerebral blood flow (rCBF). n = 3/group. Data are represented as means ± SEM. $^{***}P < 0.001$.SDN, splenic sympathetic denervation.

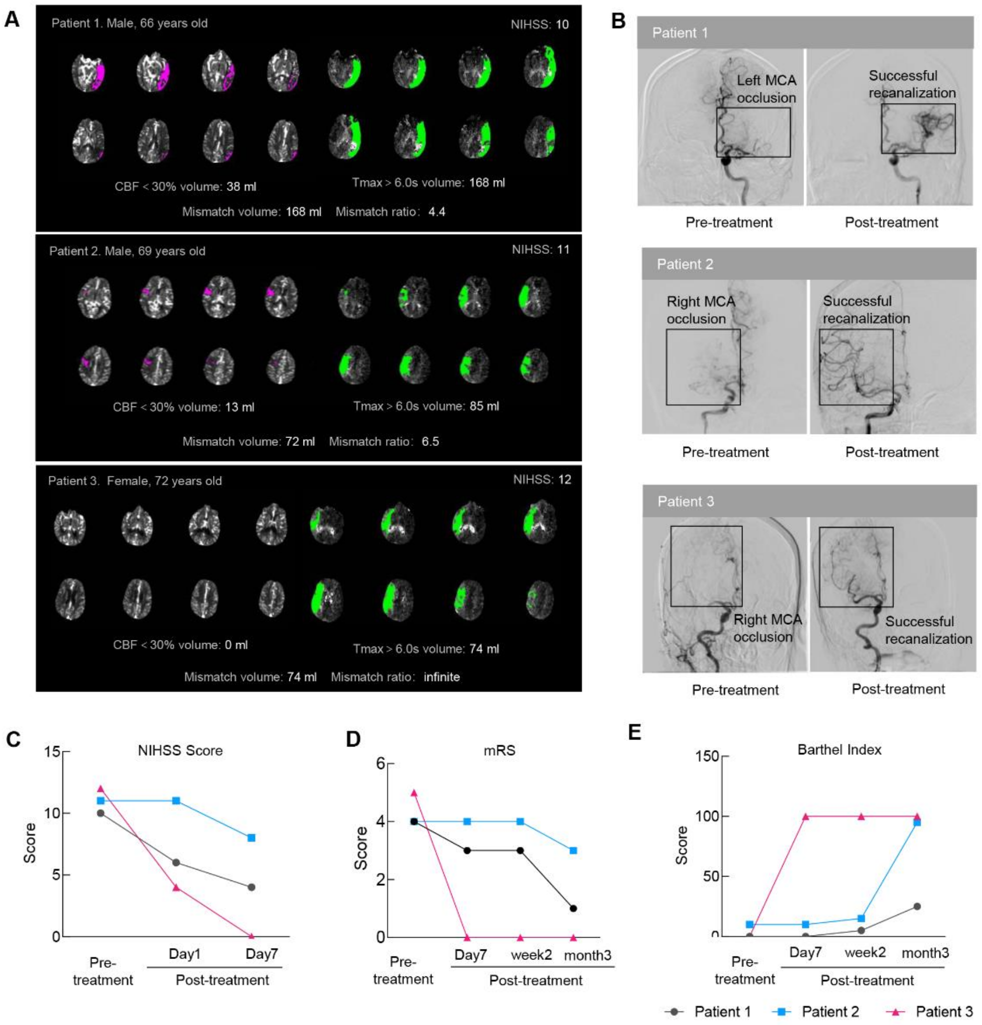

Supplementary Figure 2. Infarction and neurofunctional assessment scores of three AIS patients recruited in the scRNA-seq analysis. **A**. Representative penumbra volume was defined by computed tomography perfusion (CTP). **B.** Digital subtraction angiograms illustrating the preprocedural middle cerebral artery occlusion, followed by post-thrombectomy images demonstrating successful recanalization of its branches. **C**. NHISS scores of three AIS patients at pre-treatment, on Day 1, and on Day 7 post-treatment. **D.** mRS scores of three AIS patients at pre-treatment, on Day 7 post-treatment, 2 weeks post-treatment, and 3 months post-treatment. **E.** BI scores of three AIS patients at pre-treatment, on Day 7 post-treatment, 2 weeks post-treatment, and 3 months post-treatment.

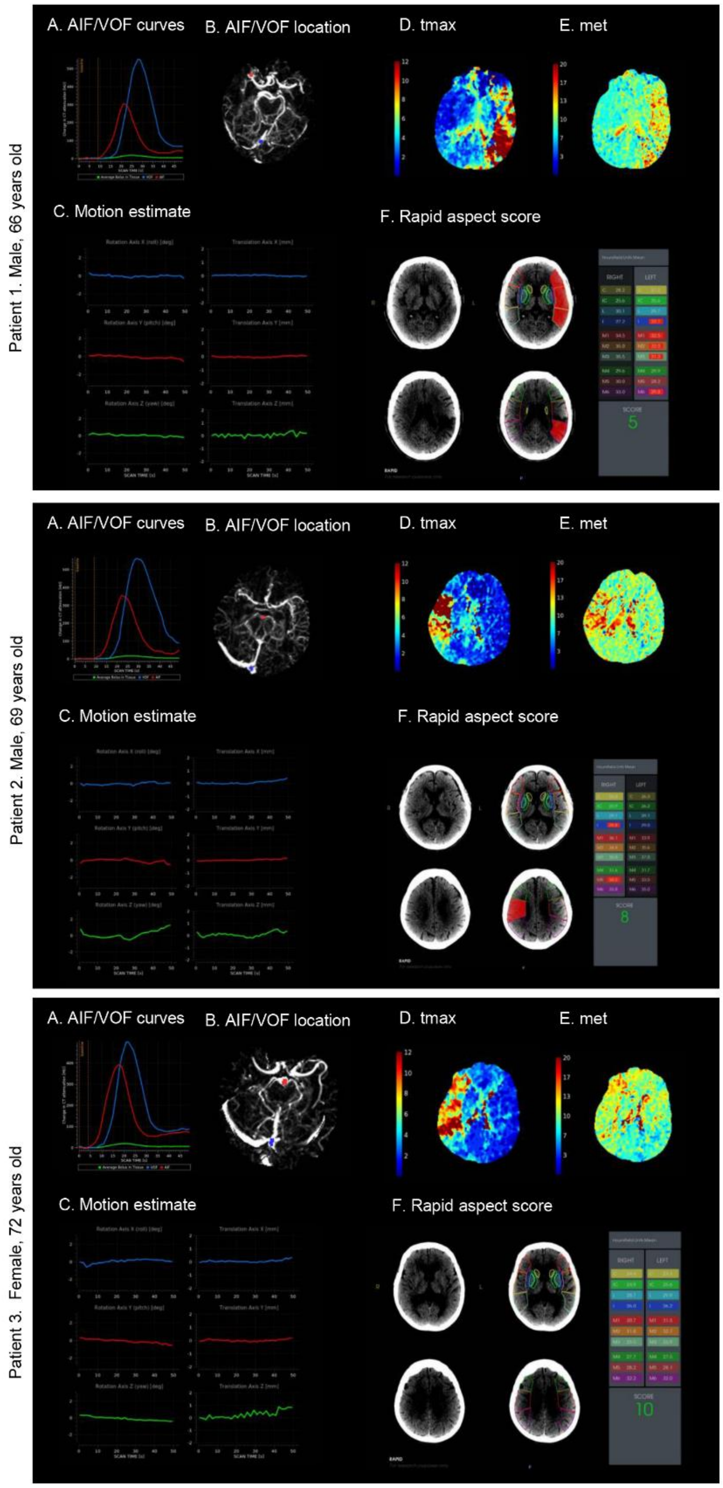

Supplementary Figure 3. Clinical characteristics of three AIS patients recruited in the scRNA-seq analysis.

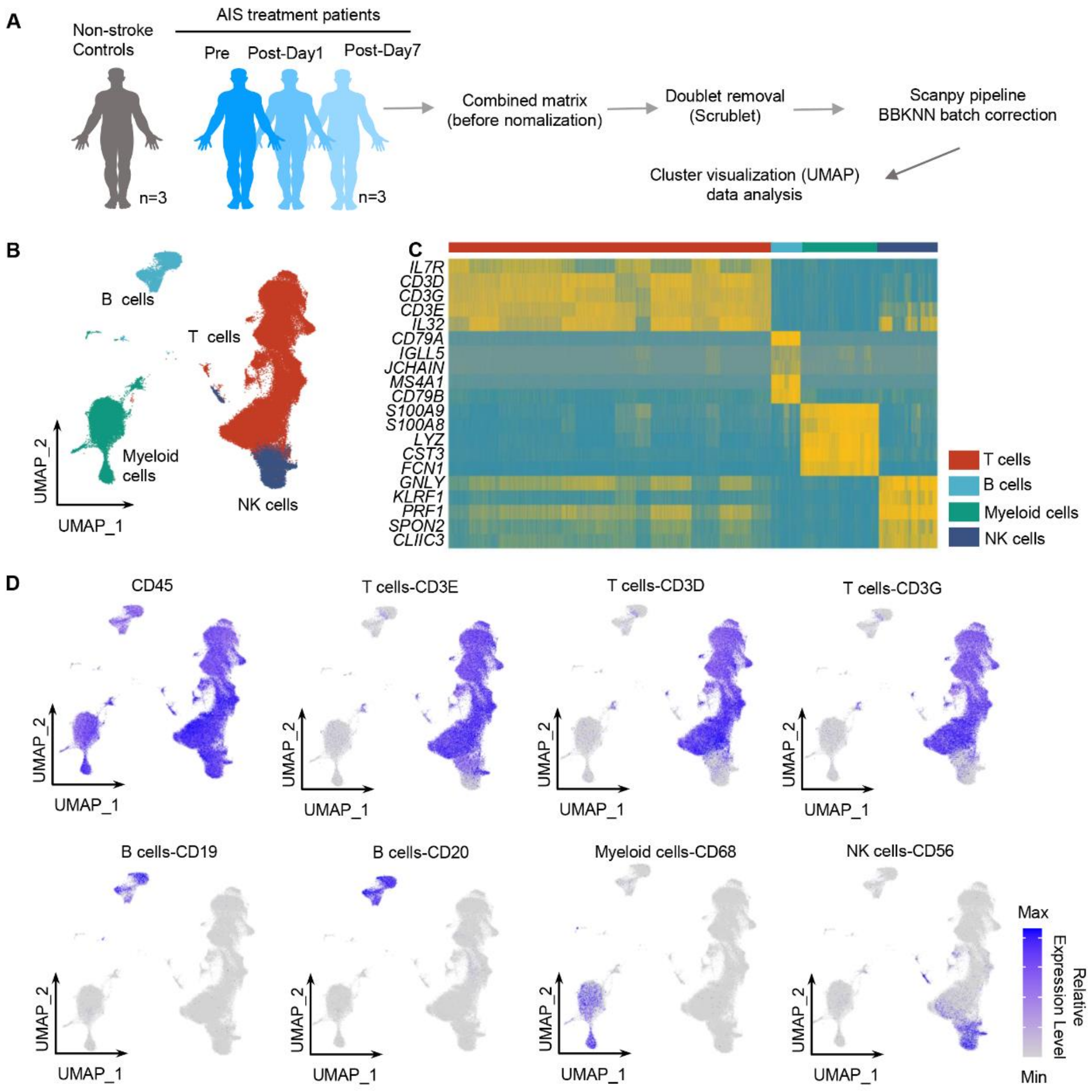

Supplementary Figure 4. scRNA-seq analysis revealed altered composition of human. A. The schematic of the scRNA-seq workflow. **B.** UMAP of the 4 major cell populations identified in the PBMCs from blood samples in non-stroke controls and AIS patients at pre-treatment, on day 1, and on day 7 post-treatment. n = 3/group. **C.** Heatmap of 5 selected genes in four defined clusters. **D.** UMAP profile of marker gene expressions in four distinct immune cell sub-clusters.

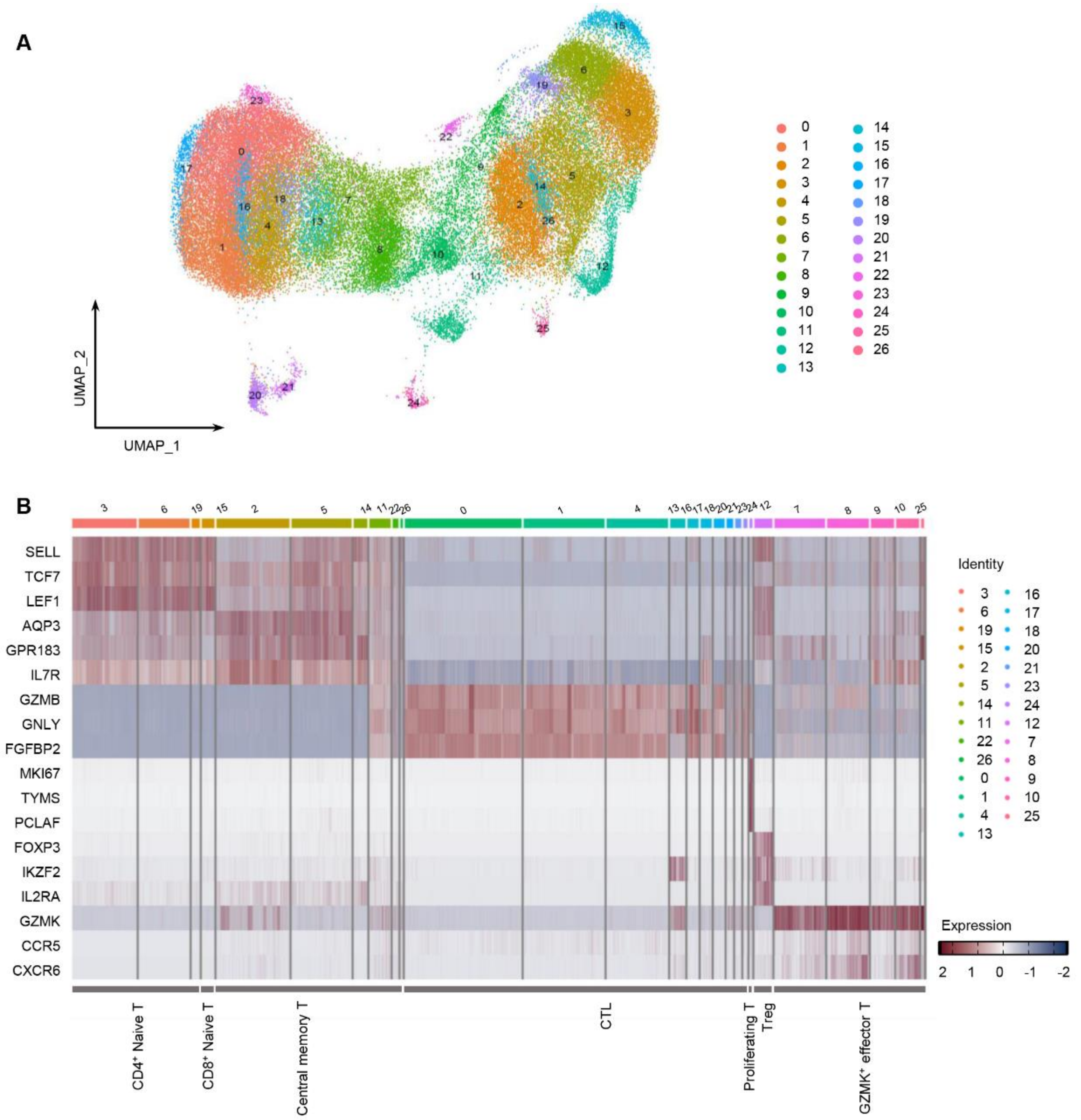

Supplementary Figure 5. scRNA-seq analysis revealed transcriptional changes of PBMCs in AIS patients. A. UMAP presentation of 27 clusters using Seurat. **B.** Heatmap of the selected marker genes in each cluster.

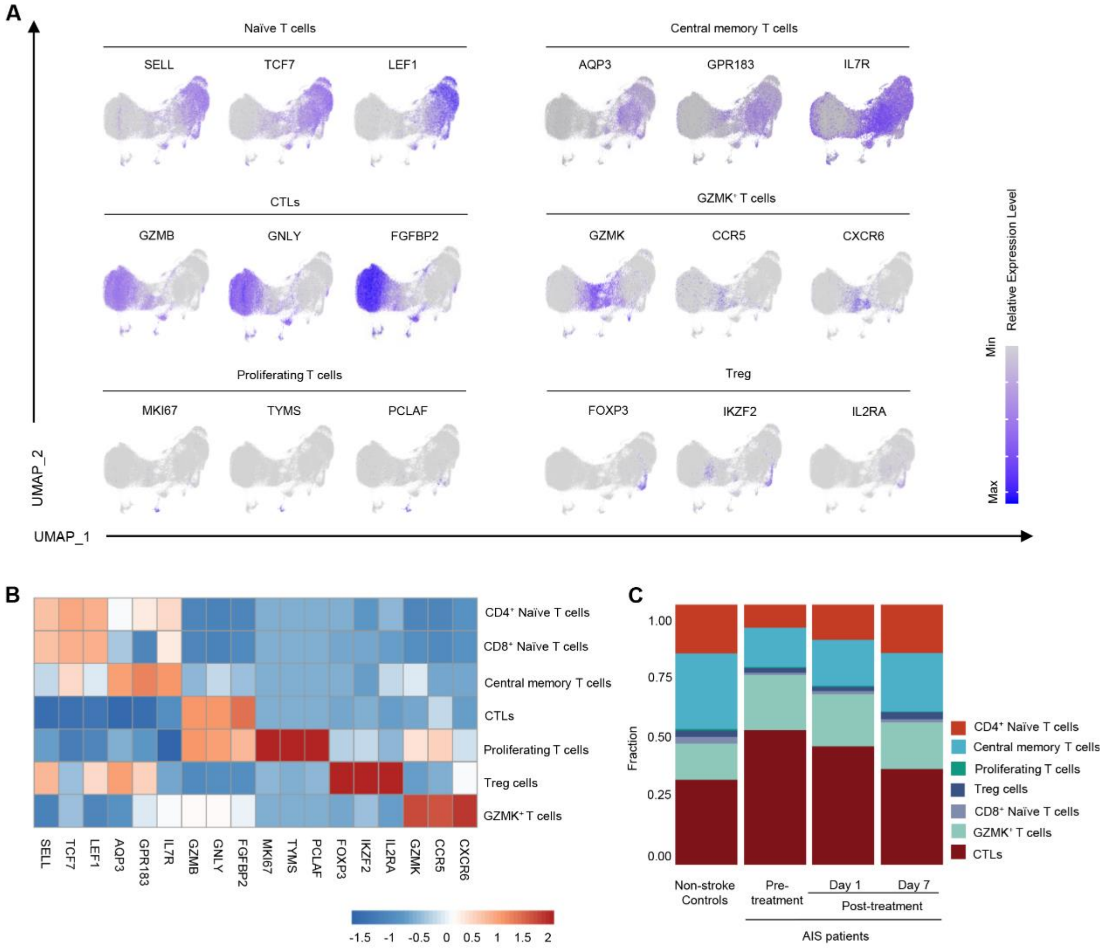

Supplementary Figure 6. Identification of T cell subsets in ischemic stroke patients. A. UMAP profile of marker gene expressions in six distinct T cell sub-clusters. **B.** A heatmap showing the hallmark genes expressed by various T cell subsets. **C.** The fractions of seven clusters defined in immune cells in each group.

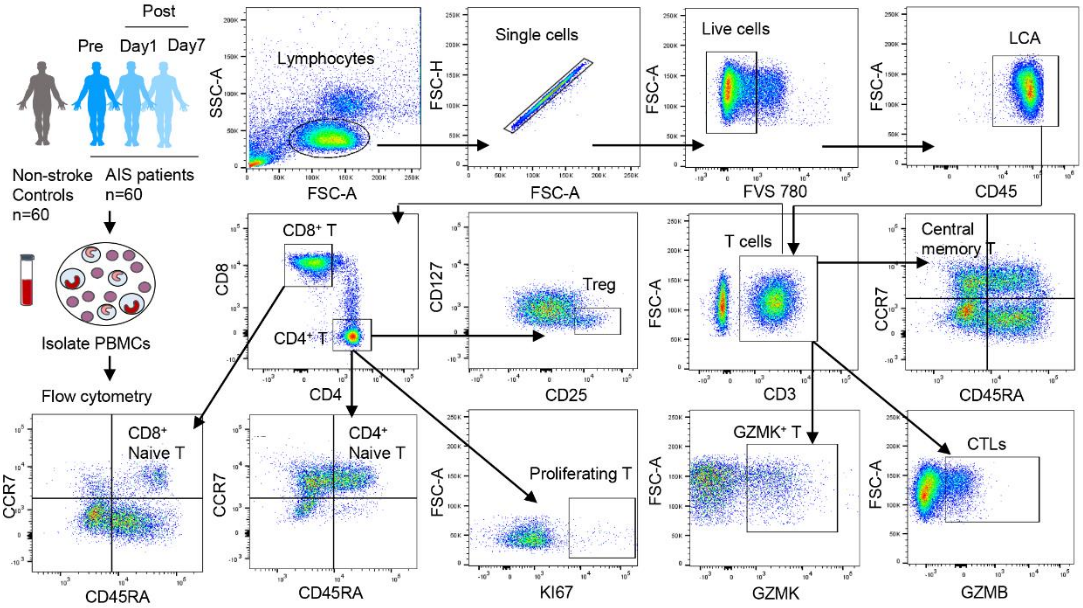

Supplementary Figure 7. The gating strategy and flow cytometry plot of T cell subsets from AIS patients and non-stroke controls. Gating strategy for determination of seven T cell subpopulation abundance by flow cytometry.

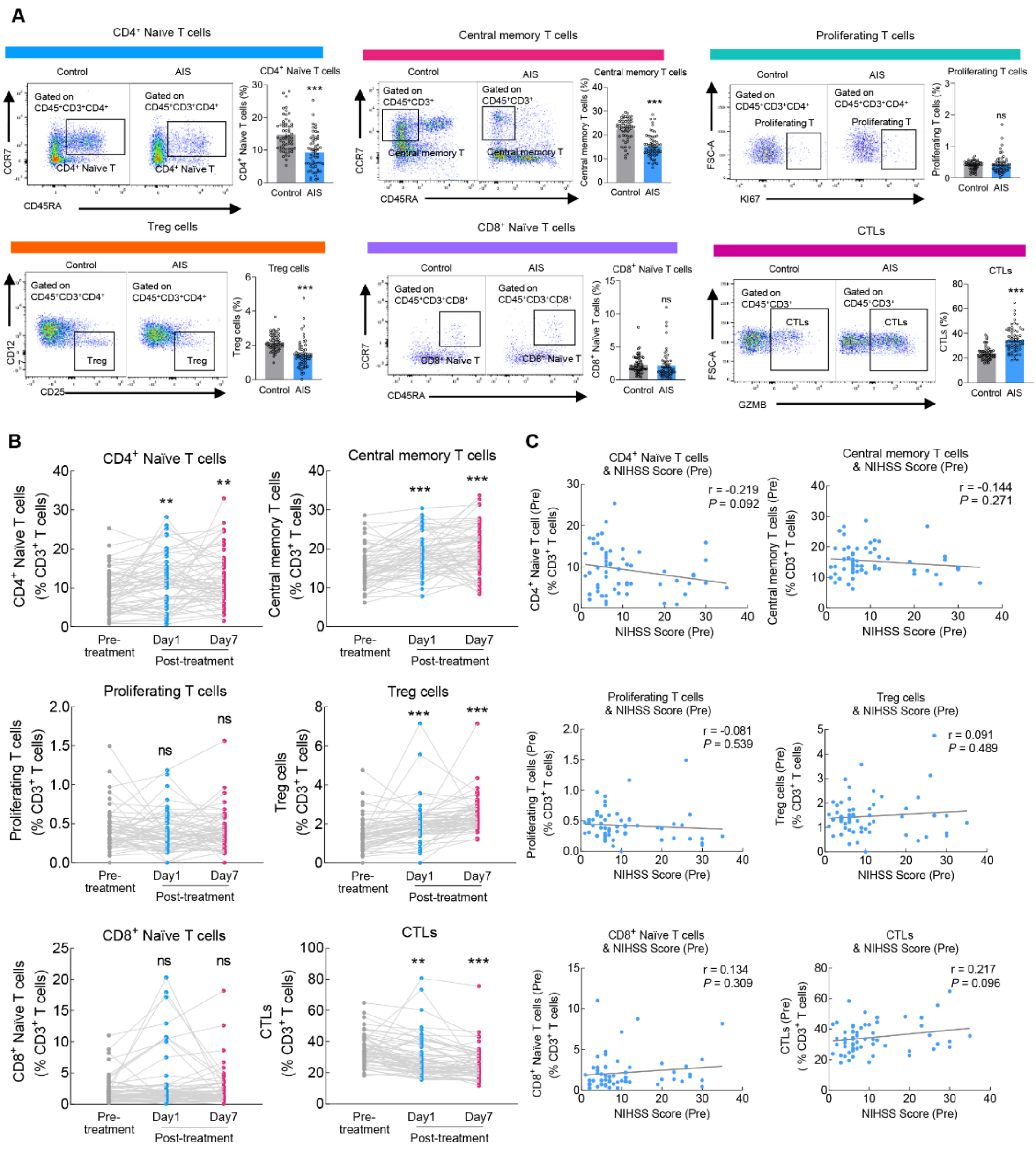

Supplementary Figure 8. Changes in the proportions of T cell subsets in the PBMCs of AIS patients. **A.** Proportions of other six distinct T cell sub-clusters in non-stroke controls and AIS patients. n = 60/group. **B.** Proportions of other six distinct T cell sub-clusters in AIS patients at pre-treatment, on day 1, and on day 7 post-treatment. n = 60/group. **C.** Spearman correlation between other six distinct T cell sub-clusters and NIHSS scores (Pre-treatment). Data are represented as means ± SEM. Pre, pre-treatment; ns, no significance; $^{**}P < 0.01$; $^{***}P < 0.001$.

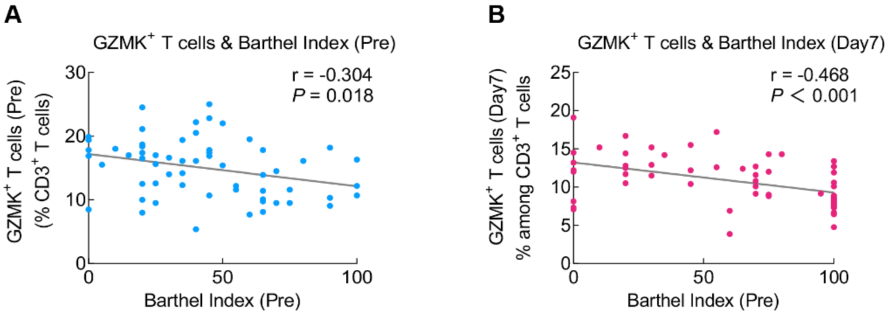

Supplementary Figure 9. Correlation between GZMK$^+$ T cell levels and stroke severity in AIS patients. A. Spearman correlation between GZMK$^+$ T cell proportions and BI scores (Pre-treatment). **B.** Spearman correlation between GZMK$^+$ T cell proportions and BI scores (Day 7 post-treatment). Pre, pre-treatment.

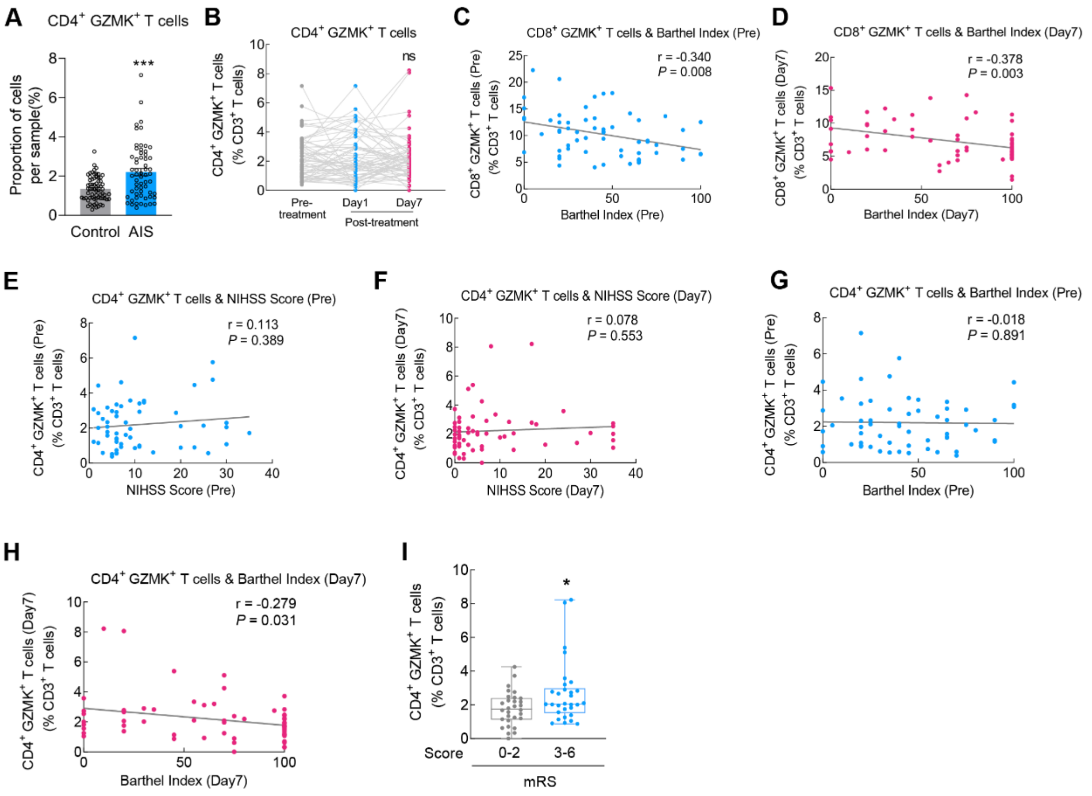

Supplementary Figure 10. Correlation between CD8^{+}/CD4^{+} GZMK^{+} T cell levels and stroke severity in AIS patients. A. Proportions of CD4^{+} GZMK^{+} T cells in non-stroke controls and AIS patients. n = 60/group. **B.** Proportions of CD4^{+} GZMK^{+} T cells in AIS patients at pre-treatment, on day1, and on day7 post-treatment. n = 60/group. **C.** Spearman correlation between CD8^{+} GZMK^{+} T cell proportions and BI scores (Pre-treatment). **D.** Spearman correlation between CD8^{+} GZMK^{+} T cell proportions and BI scores (Day7 post-treatment). **E.** Spearman correlation between CD4^{+} GZMK^{+} T cells proportions and NHISS scores (Pre-treatment). **F.** Spearman correlation between CD4^{+} GZMK^{+} T cell proportions and NHISS scores (Day 7 post-treatment). **G.** Spearman correlation between CD4^{+} GZMK^{+} T cell proportions and BI scores (Pre-treatment). **H.** Spearman correlation between CD4^{+} GZMK^{+} T cell proportions and BI scores (Day 7 posttreatment). **I.** Proportions of CD4^{+} GZMK^{+} T cells in patients with good outcomes (mRS 0–2) versus poor outcomes (mRS 3–6) on day 1 versus day 7 post-treatment; n = 30 in the good outcome group and n = 30 in the poor outcome group. Data are represented as means ± SEM. Pre, pre-treatment. ns, no significance; $^{*}P < 0.05$; $^{***}P < 0.001$.

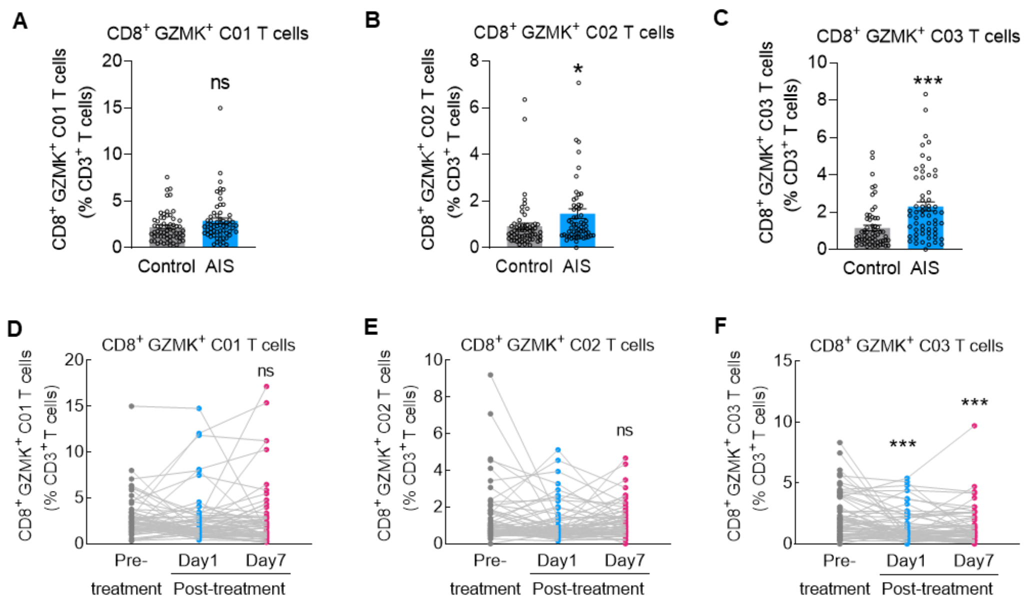

Supplementary Figure 11. Changes of the different CD8$^+$ GZMK$^+$ T cell subsets in AIS patients. **A-C.** Proportions of CD8$^+$ GZMK$^+$ C01, C02, and C03 T cells in non-stroke controls and AIS patients. n = 60/group. **D-F.** Proportions of CD8$^+$ GZMK$^+$ C01, C02, C03 T cells in AIS patients at pretreatment, on day 1, and on day 7 post-treatment. n = 60/group. Data are represented as means ± SEM. ns, no significance; $^{*}P < 0.05$; $^{***}P < 0.001$.

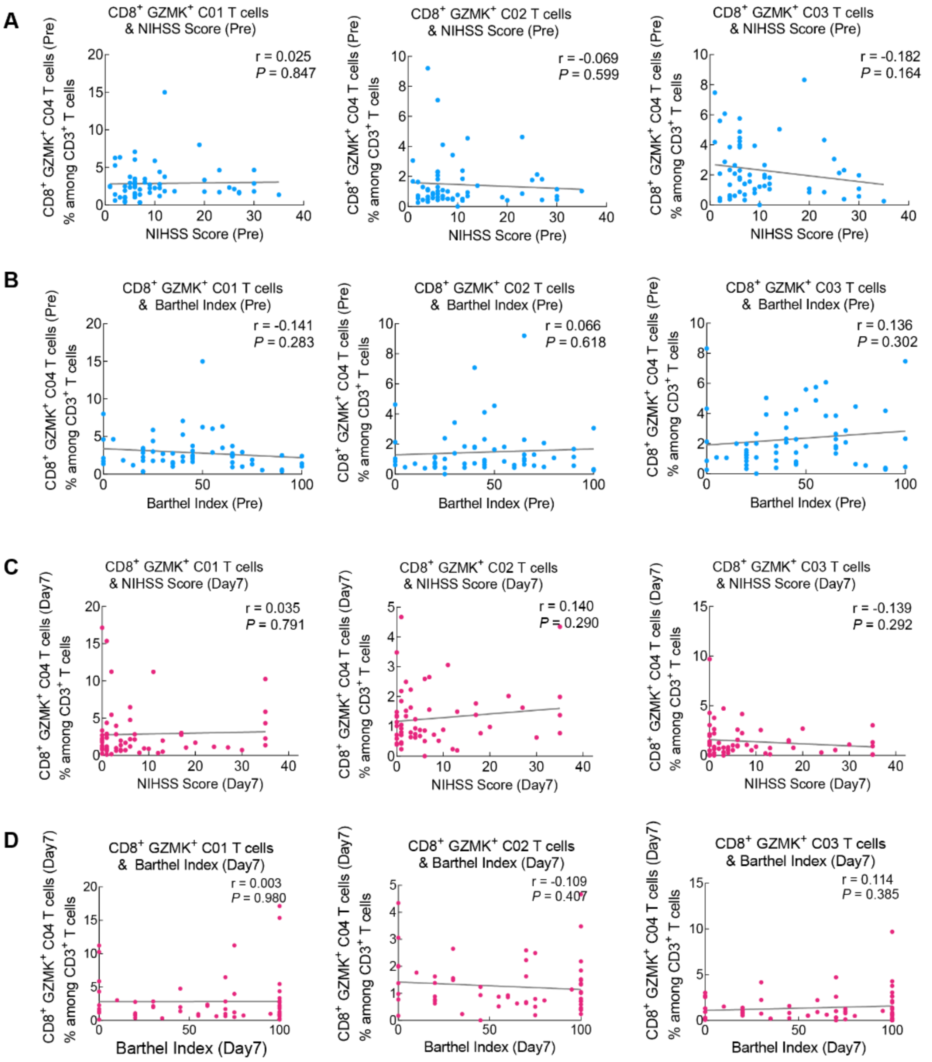

Supplementary Figure 12. Correlation between CD8$^+$ GZMK$^+$ T cell subsets and stroke severity in AIS patients. A. Spearman correlation between CD8$^+$ GZMK$^+$ C01, C02, and C03 T cell proportions and NHISS scores (Pre-treatment). **B.** Spearman correlation between CD8$^+$ GZMK$^+$ C01, C02, and C03 T cell proportions and BI scores (Pre-treatment). **C.** Spearman correlation between CD8$^+$ GZMK$^+$ C01, C02, and C03 T cell proportions and NHISS scores (Day 7 post-treatment). **D.** Spearman correlation between CD8$^+$ GZMK$^+$ C01, C02, and C03 T cell proportions and BI scores (Day 7 post-treatment). Pre, pre-treatment.

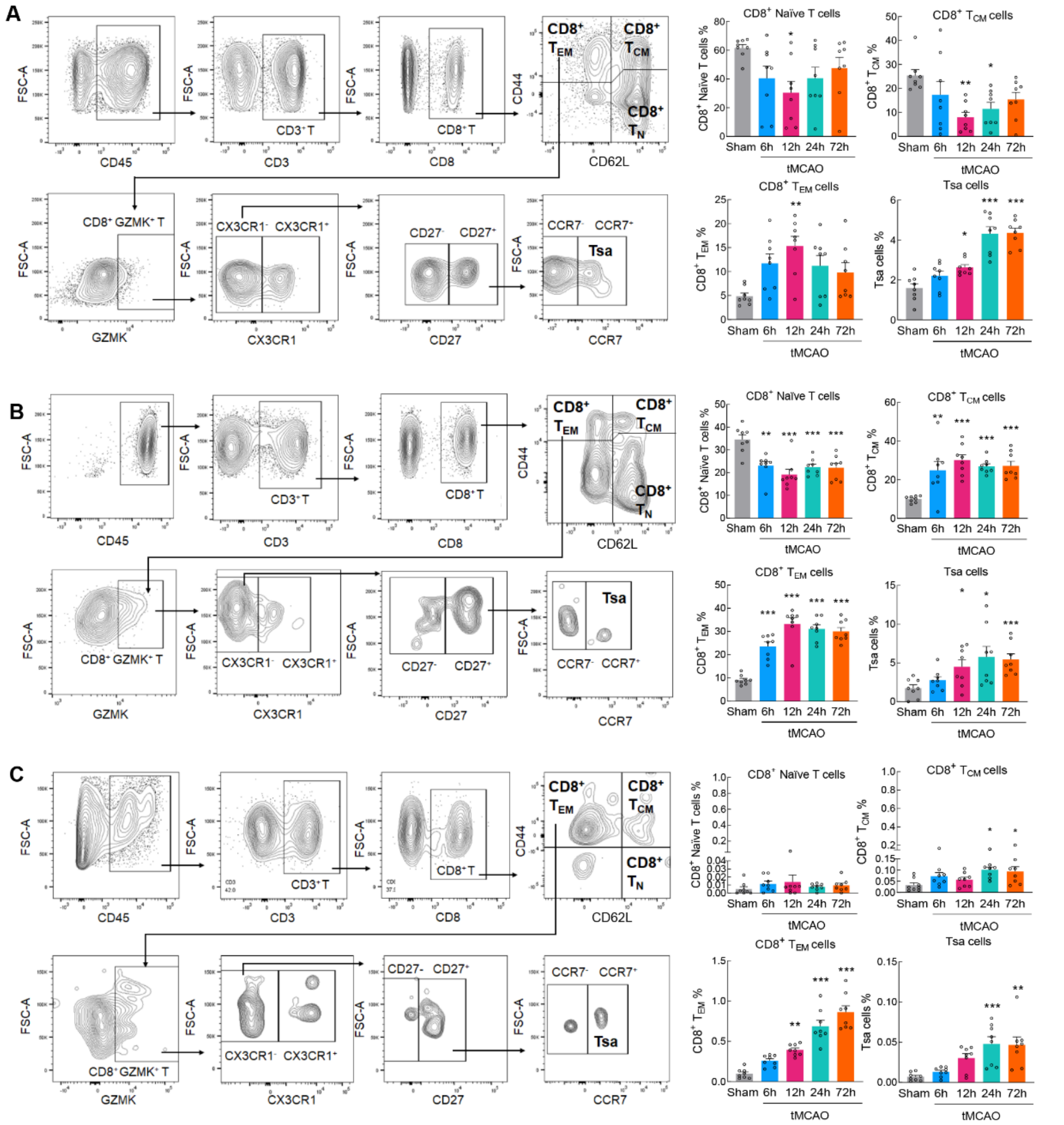

Supplementary Figure 13. Time course of CD8⁺ Naive T cells, CD8⁺ T$_{CM}$ cells, CD8⁺ T$_{EM}$ cells and Tsa cells after tMCAO. A-C. Bars graph show the proportions of CD8⁺ Naive T cells, CD8⁺ T$_{CM}$ cells, CD8⁺ T$_{EM}$ cells and Tsa cells in the spleen **(A)**, blood **(B),** and brain **(C)** detected by flow cytometry at 6 h,12 h,24h and 72 h after reperfusion. n = 8/group. Data are represented as means ± SEM. $^{*}P< 0.05$; $^{**}P < 0.01$; $^{***}P < 0.001$.

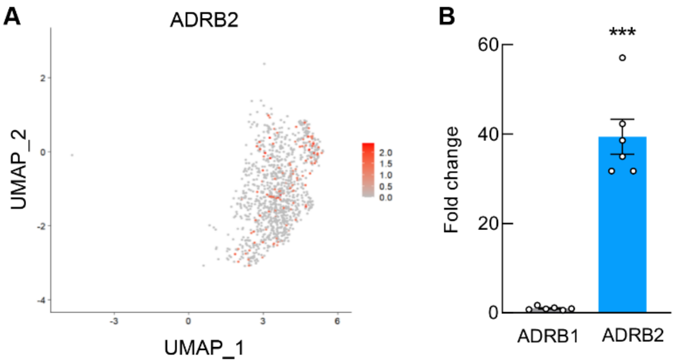

Supplementary Figure 14. The specific neurotransmitter or signaling pathways of Tsa cells. A. Expression level of ADRB2 in the Tsa cells overlaid on the UMAP representation. **B.** Relative expressions of ADRB1 and ADRB2 in the sorted Tsa cells as determined by qPCR. n = 6/group. Data are represented as means ± SEM. $^{***}P < 0.001$.

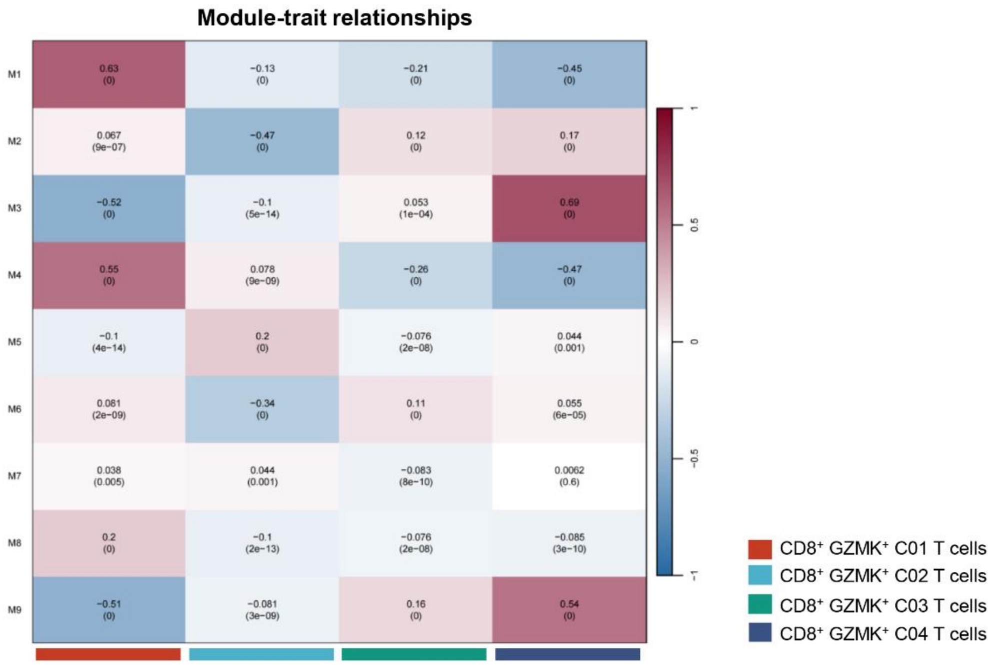

Supplementary Figure 15. Weighted correlation network analysis (WGCNA) of CD8^{+} GZMK^{+} T cell subtypes. Heatmap showing Pearson correlation between CD8^{+}GZMK^{+} T cell subtypes and the eigengenes of gene co-expression modules defined by WGCNA in all CD8^{+} GZMK^{+} T cells.

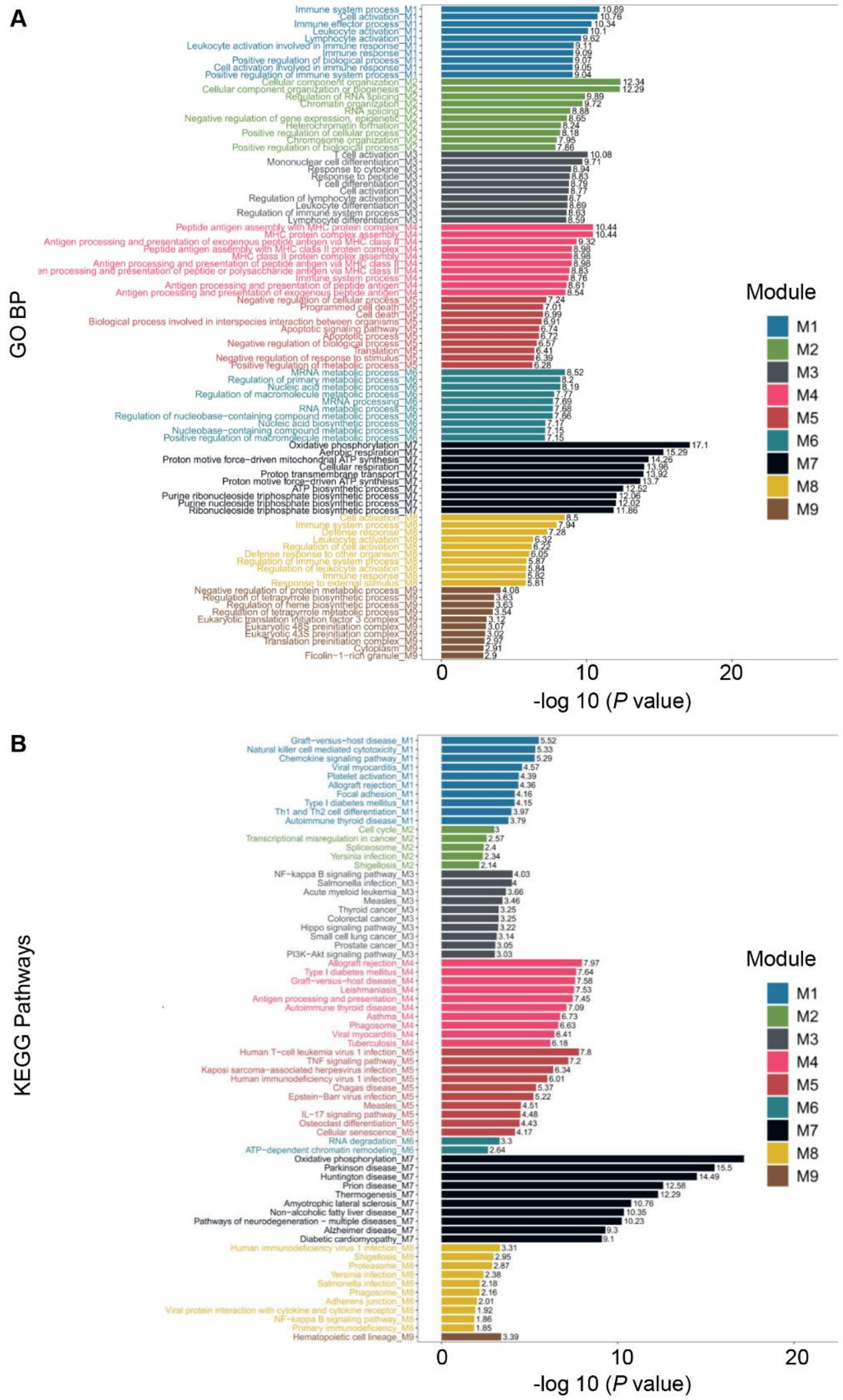

Supplementary Figure 16. GO and KEGG analysis of of CD8$^+$ GZMK$^+$ T cell subtypes. A. Top gene ontology terms enriched in each gene co-expression module in CD8$^+$ GZMK$^+$ T cells. **B.** KEGG enriched in each gene co-expression module in in CD8$^+$ GZMK$^+$ T cells.

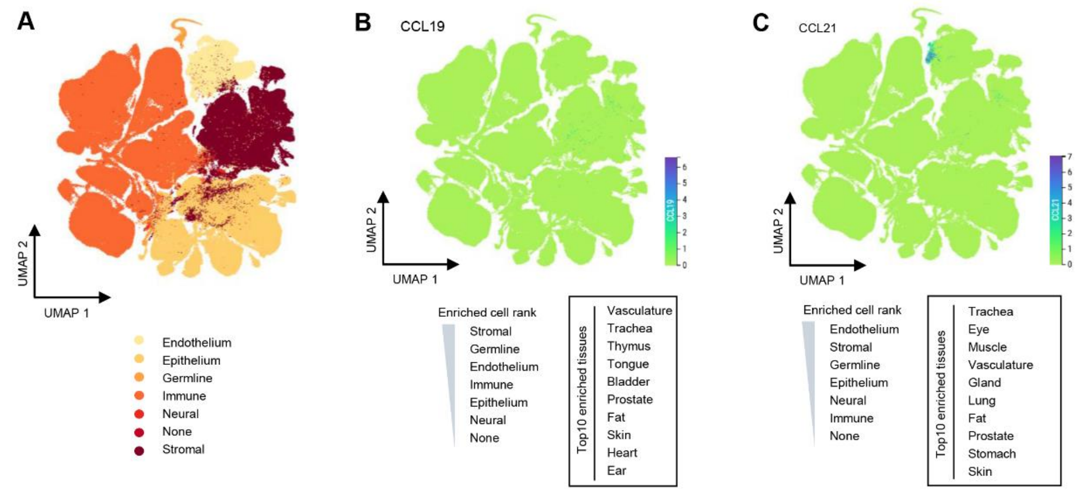

Supplementary Figure 17. Cross-tissue analysis using Tabula Sapiens Consortium data. A. Cross-tissue analysis using Tabula Sapiens Consortium data for CCR7 ligands. **B.** Cross-tissue analysis for CCL19. **C.** Cross-tissue analysis for CCL21.

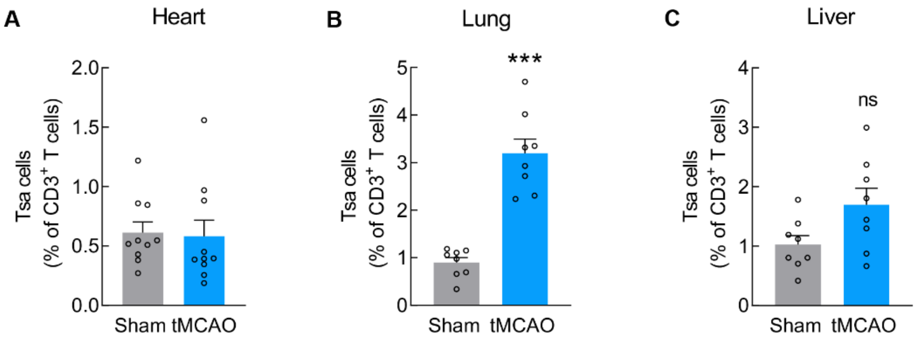

Supplementary Figure 18. Proportions of Tsa cell in heart, lung and liver of tMCAO mice. A-C. Proportions of Tsa cell in heart **(A)**, lung **(B)** and liver **(C)** as examined by flow cytometry. Data are represented as means ± SEM. ns, no significance; $^{***}P < 0.001$.

A

CCR7 peptide	Sequence	Molecular Weight (g/mol)
CCR7 peptide 28-33	VTDDYI-NH_2	723.77
CCR7 peptide 29-35	TDDYIGD-NH_2	796.77

B

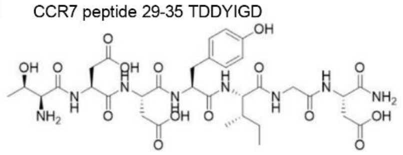

C

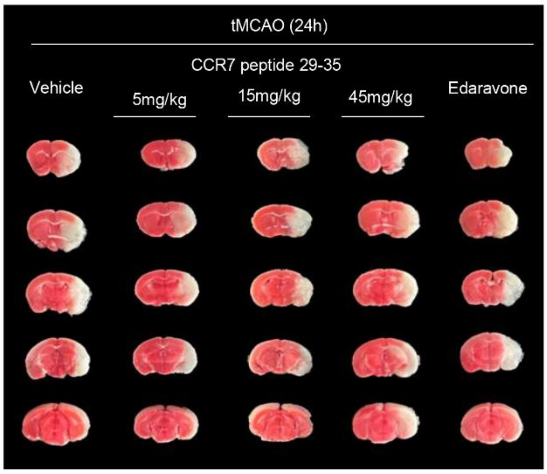

D

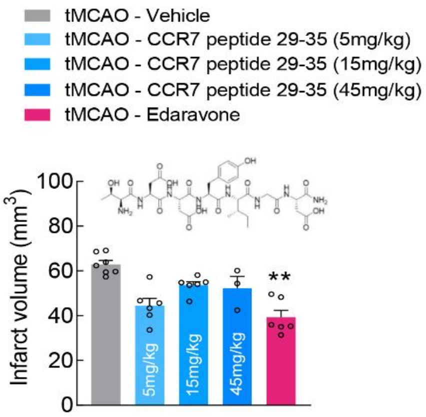

Supplementary Figure 19. CCR7 peptide 29-35. A. Sequence and molecular weight of two candidate peptides (CCR7 peptide 28-33 and CCR7 peptide 29-35). **B.** Potential peptide fragments of the CCR7 peptide 29-35. **C-D.** Representative TTC stained sections 24 hours after tMCAO in the Vehicle, three different dosages of CCR7 peptide 29-35 (5 mg/kg, 15 mg/kg, and 45 mg/kg), and Edaravone groups, followed by an analysis of infarct volume. n = 3-8/group. Data are represented as means ± SEM. $^{**}P < 0.01$.

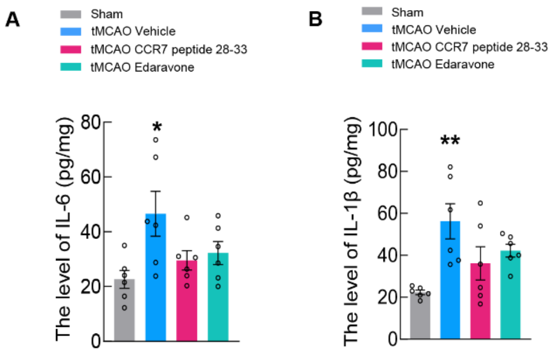

Supplementary Figure 20. The expression of IL-6 and IL-1β. A-B. Level of IL-6 **(A)** and IL-1β **(B)** measured by ELISA in the brain 24 hours after tMCAO in the sham, Vehicle, CCR7 peptide 28-33 (15 mg/kg) and Edaravone groups. n = 6/group. Data are represented as means ± SEM. $^{*}P < 0.05$; $^{**}P < 0.01$.
