# Supplementary figures and images for: CD8+GZMK+CD27+CCR7+ T cells mobilized by splenic sympathetic nerves aggravate brain ischemia‒reperfusion injury via CCL19-positive endothelial cells
Source: Cell Mol Immunol. 2025 Jul 14;22(9):1061–76. doi: 10.1038/s41423-025-01311-9 (PMC12398583; doi:10.1038/s41423-025-01311-9)

**Supplementary information-Original western blot images**

**
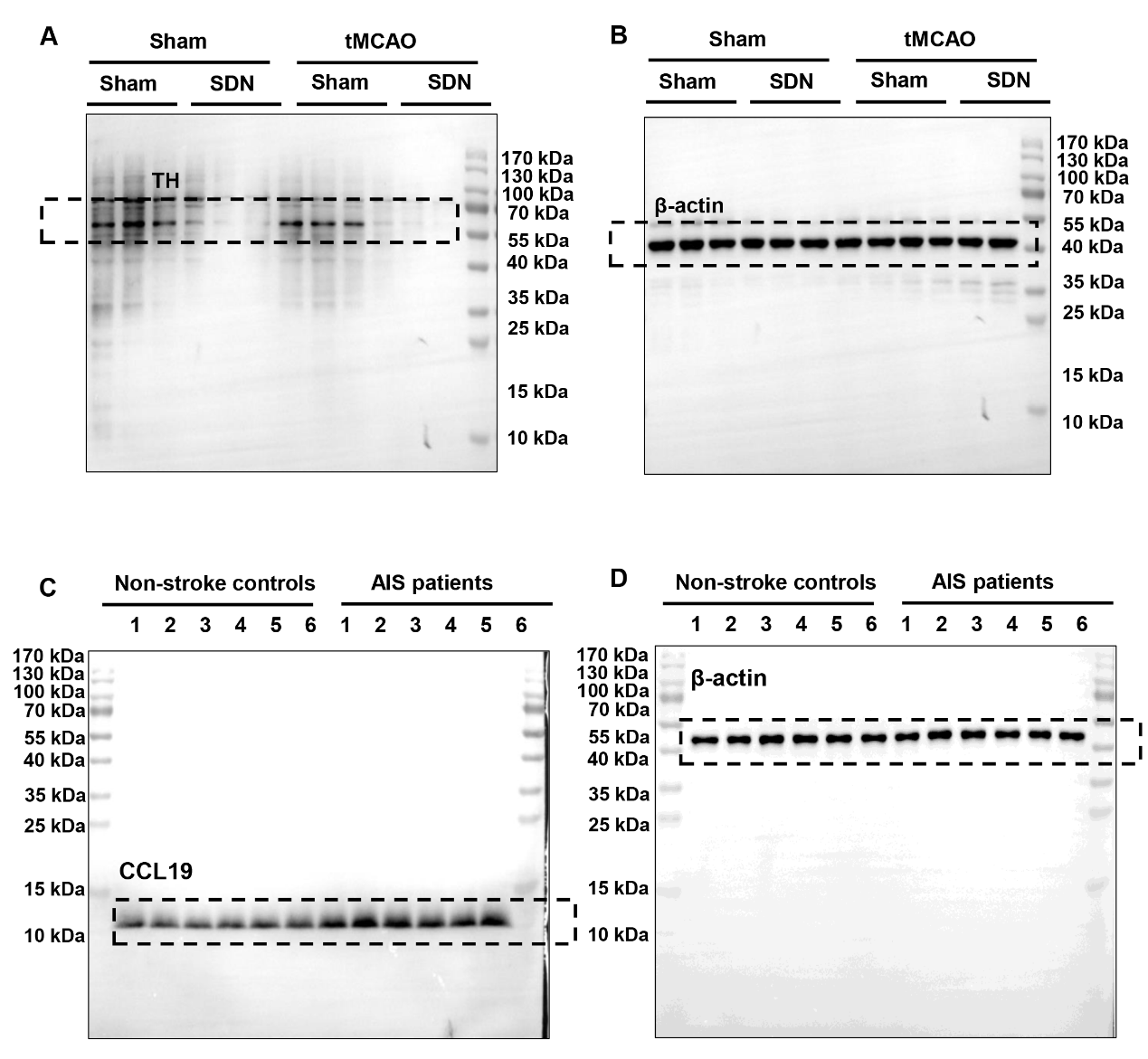
**

Supplement: Supplementary file 3 — Supplementary information-Original western blot images [file 41423_2025_1311_MOESM3_ESM.docx]
